# Synthesis, spectroscopic characterization and dyeing performance of novel bis azo dyes derived from benzidine

**DOI:** 10.1038/s41598-023-34660-4

**Published:** 2023-05-15

**Authors:** Alaa Z. Omar, Mohamed A. El-Rahman, Ezzat A. Hamed, Samir K. El-Sadany, Mohamed A. El-atawy

**Affiliations:** 1 Chemistry Department, Faculty of Science, Alexandria University, P.O. 426, Ibrahemia, Alexandria, 21321 Egypt; 2 Chemistry Department, Faculty of Science, Taibah University, Yanbu, 46423 Saudi Arabia

**Keywords:** Chemistry, Organic chemistry, Theoretical chemistry

## Abstract

Benzidine was coupled with ethyl cyanoacetate, and malononitrile, to give azo-hydrazo products which in turn were cyclized by using hydrazine and phenyl hydrazine to give 4,4'-([1,1'-biphenyl]-4,4'-diylbis(hydrazin-2-yl-1-ylidene))bis pyrazole derivatives **5–7**. These compounds were identified by various spectral analysis. The examination of 0.1 M NaOH and 0.1 M HCl in DMF revealed that the λ_max_ of the synthesized dyes are quite sensitive to pH variation and slightly affected by the coupler moieties. Utilizing the dispersion agent DYEWELL-002, polyester fabric (PE-F) was dyed in water. The color strength (K/S), its summation (K/Ssum), dye exhaustion (%E) and reflectance values were measured and discussed. The DFT method estimates the chemical descriptor parameters of the titled dyes, using B3LYP/6-31G(d,p) level to investigate the performance of dyes as well as to postulate a mechanism of dyeing process.

## Introduction

Azo dyes are the largest class of commercial dyes due to their presence in most classification of dyes. These compounds are identified by the presence of a chromophoric group (–N = N–) as in acid, reactive, azoic, direct, and basic dyes. Generally, the azo chromophore is bonded to aromatic derivative rings, naphthalene ring derivatives, aromatic heterocycles or replaces an acidic hydrogen atom of an active methylene group^1^. Such a group must flank the azo group to provide the dye color with their hues of various intensities. The importance of azo dyes came from their use as synthetic colorants in textile, printing, paper manufacturing, etc.^2^. Additionally, azo substances are used for biocidal treatment of textile materials, such as antiseptics, antineoplastics, antidiabetics and antitumor because azo dyes exhibit biological and medicinal importance^3–5^. Furthermore, many research and studies on azo dyes have been conducted to introduce or to modify new characteristics. This is a result of enhanced thermal and optical properties of azo dyes^2,6–9^. To create bisazo dyes, an aromatic diamine, such as benzidine, is typically diazotized twice and linked via the same or different couplers Benzidine-based azo dyes and congener-based dyes are used in the production of paints, printing inks, pharmaceuticals^10^, and in the food, petroleum, fur, plastics, hair dyes, soap, rubber, and woods industries^11,12^. Azo dyes derived from benzidine are classified as a source of pollution that affect the environment^13^. However, there is no significant proof that the orange and yellow pigments produced from benzidine namely disazopyrazolones (diarylide oranges) pigments themselves pose a hazardous danger^14,15^. Most azo dyes are reported to azo-hydrazo tautomerism, Therefore, there are several studies on azo dye tautomerism in the literature, which pertain to sectors including optical, technological, and environmental applications^16,17^. The pH of the medium, solvent polarity, and structural variables are the decisive factors of the tautomeric equilibrium. Additionally, some researchers have used calculations based on density functional theory (DFT) to study the tautomerism of azo dyes^18^. The azo benzidine-based pyrazole rings are structurely similar to that of tartrazine (C.I. Food Yellow 4), a synthetic lemon yellow azo dye primarily used as a food coloring. This similarity suggests that there may be shared properties or behaviors between the two compounds, such as color stability or reactivity.

In continuation of our interest in the field of dyes^19,20^, this work is aimed to examine the structure performance relationship of various bisazo dyes that are produced by coupling with active methylene molecules, namely malonoinitrile, and ethyl cyanoacetate with diazonium salts derived from benzidine. These diazotized compounds undergo cyclization with hydrazine hydrate and phenyl hydrazine to form pyrazole rings at the two ends of the molecule. The spectral analysis of the synthesized azo dyes in this work are measured experimentally and calculated by using DFT method with the basis set of 6-31G(d,p) to determine their molecular structure. The possibility of tautomerism of the synthesized azo dyes and the global parameters using density functional theory (DFT) calculations are examined to investigate parameters affecting the dye ability of these dyes as well as suggesting a possible mechanism of dyeing. To verify the dyeability of the synthesized bis azo dyes, the fastness properties such as light, washing, perspiration, and heat fastness were examined on polyester fabrics.

## Results and discussion

### Chemistry

**Figure 1:**
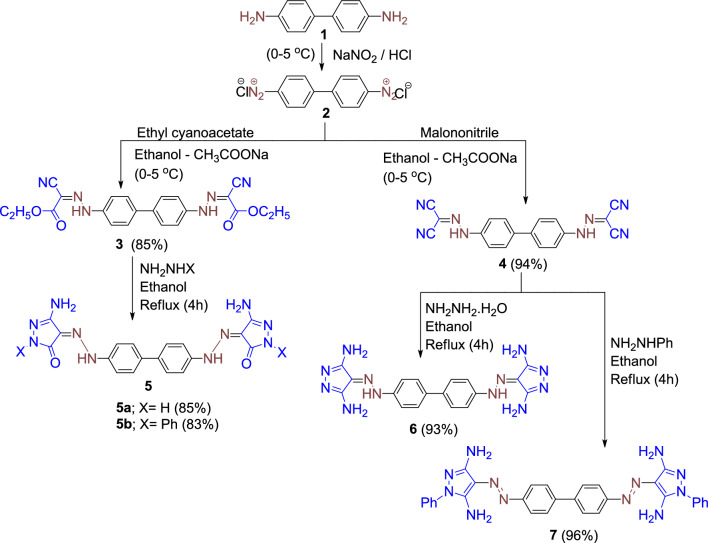
The possible synthetic pathways to dyes **3–7**.

Starting with benzidine **1**, bis azo dyes **3–4**, were synthesized by diazotizing it with a solution of sodium nitrite in diluted hydrochloric acid at 0 °C to produce 1,1'-biphenyl-4,4'-bis(diazonium) chloride **2.** This was followed by coupling with ethyl cyanoacetate, and malononitrile, (both containing an active methylene group) in the presence of sodium acetate gave the insoluble disperse dye products diethyl 2,2'-([1,1'-biphenyl]-4,4'-diylbis(hydrazin-2-yl-1-ylidene))-bis(2-cyanoacetate) **3**, and *N*',*N*''-([1,1'-biphenyl]-4,4'-diyl)dicarbonohydrazono -yl dicyanide **4.** Cyclization of products **3–4** by the action of hydrazine hydrate (98%) and phenylhydrazine to give the pyrazole dyes **5–7** as a type of heterocyclic bis azo dyes, Fig. 1.

**Figure 2:**
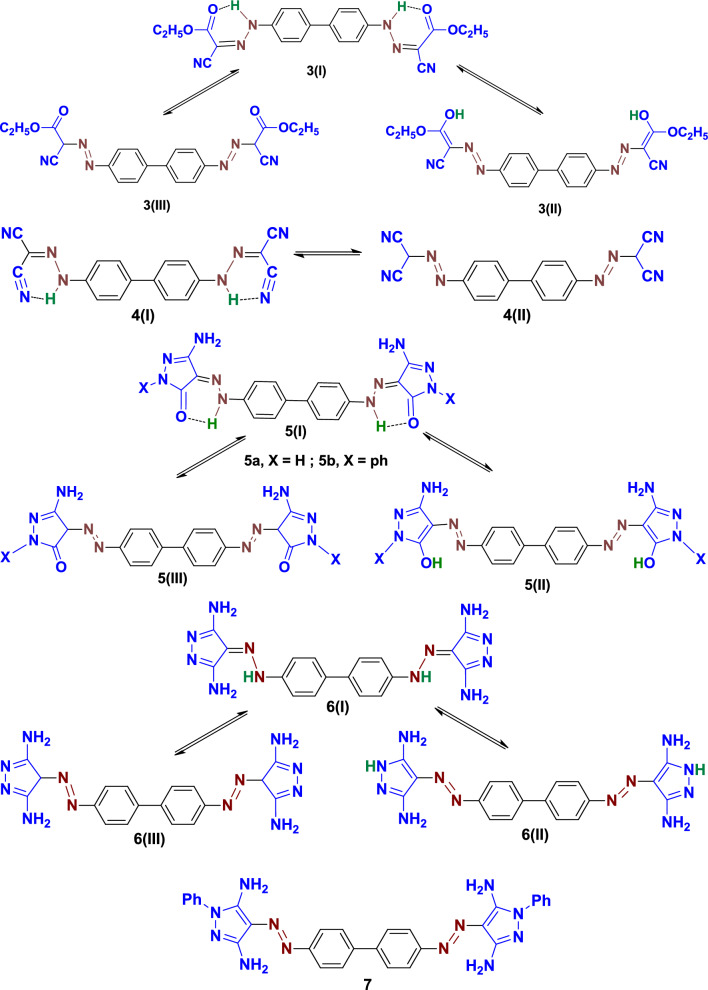
Possible tautomeric forms of compounds **3–7**.

The various properties of azo-hydrazo tautomerism can result in diverse characteristics such as biological, photo-physical, technical and thermal applications^17,21^. Compounds **3**, and **5a,b** can exist in three possible tautomeric forms, namely, the hydrazo-carbonyl form **3(I), 5(I)**, azo-enol form **3(II), 5(II)** and azo-carbonyl form **3(III), 5(III).** Compound **4** exists in two tautomeric forms, the hydrazo **4(I)** and its azo tautomer **4(II)**, whereas, the three possible tautomeric forms of compounds** 6** are the hydrazo form **6(I)** and two azo forms, **6(II)** and **6(III)**. Finally, only one tautomer, namely azo form, is postulated to compound **7** due to the absence of acidic proton. The tautomeric forms of compounds **3–7** are illustrated in Fig. 2.

The structures of the azo-hydrazo compounds **3–7** were identified and characterized by UV–visible, FTIR, ^1^H NMR and ^13^C NMR spectroscopy, while a thin layer chromatography (TLC) was used to test the purity of products**.**

The IR^22^ spectra in the solid phase of bis azo dyes **3–6** showed peaks at ʋ 3414–3182 cm^-1^ corresponding to N–H stretching vibrations, consistent with their existence in the hydrazo tautomer. Additionally, the carbonyl stretching frequencies of the dyes **3** and** 5** played a significant role in the formation of intramolecular hydrogen bonding. According to structures **3(I)** and **5(I)**, the low stretching values of carbonyl and hydrazo NH groups point to the existence of intramolecular hydrogen bonding.

The intense peak at ʋ 1733 cm^− 1^ corresponds to ester carbonyl groups (C = O) **3,** shifted to lower frequency (1636–1623 cm^− 1^)^23^ due to the conversion to amide carbonyl groups (C = O) of pyrazolinone ring by the action of hydrazine and phenylhydrazine on dye **3** forming dyes **5a** and **5b,** respectively**.** The IR spectra of dyes **3** and** 4** showed an intense peak at υ 2210–2220 cm^− 1^ due to cyano groups vibrations. The disappearance of the –CN stretching vibration in IR spectra of compounds **3** and **4** and the presence of NH_2_ stretching vibration at ʋ 3419–3366 cm^− 1^ in dyes **5–7** also confirmed the formation of pyrazole rings. The weak peak at ʋ 1552 cm^− 1^ for azo group (N = N) is assigned to dye **7**. The observed medium peak at υ 1625–1539 cm^− 1^ of dyes **3–7** is assigned to imino (C = N), while dyes **3–7** showed a weak peak at υ 3064–3010 cm^− 1^ due (C–H sp^2^) stretching vibration and weak peak at υ 2988 cm^− 1^ for (C–H sp^3^) of dye **3.**

According to the IR measurement in the solid phase, dyes **3–5** lacked the –OH peak at 3368–3401 cm^− 1^, and the appearance of the peak at ʋ 3414–3182 cm^− 1^ corresponding to NH groups of the hydrazo moiety indicate that the dyes **3** and** 5** exist in the hydrazo form **3(I)**, and **5(I),** respectively. The absence of NH peak and the appearance weak peaks at ʋ 1563 cm^− 1^ for the azo group (N = N) in dye** 7**, indicate its existence in the azo form **7.**

An initial clue for assigning the tautomeric state was provided by the ^1^H NMR spectra, which demonstrated conclusively that a range of newly dyes exist in solution exclusively in the hydrazo, azo or an equilibrium mixture of several tautomeric species and definitive assignments of all the signals in the spectra were made. At first, the ^1^H NMR spectral data do not show any more high field signals around 5.5 ppm for CH of tautomeric forms **3(III), 4(II), 5(III)** and **6(III)** of the annulated similar compounds, respectively^24,25^. Therefore, these tautomers are hardly formed because of their low stabilities due to the less degree of conjugation.

The ^1^H NMR spectra of bis azo dyes **3–6** in DMSO-*d*_*6*_ (solution phase) showed highly deshielded protons in the range δ 13.13–10.79 ppm, attributable to two NH protons of hydrazo tautomers, except dye **7**. It was reported that the hydrazo NH proton resonance often develops between 12.0 and 15.0 ppm^26^.

The appearance of exchangeable broad singlet signals at δ 12.99–10.55 ppm in the downfield region for dye **5a**, are assigned to NH protons of the of pyrazole ring moieties. The ^1^H NMR of dyes **3–7** exhibited two doublet signals in the range δ 7.97–7.54 ppm, assigned to the aromatic protons of the biphenyl moiety. The ^1^H NMR spectra exhibited signals in the range δ 7.73–7.11 ppm, assigned to phenyl ring moieties for dyes **5b** and** 7**. Compound **3** showed a highly shielded proton at δ 1.32 ppm, attributable to CH_3_ protons and a quartet signal at δ 4.31 for the methylene group. The measurement of IR in the solid phase and the ^1^HNMR in the solution phase showed that all dyes under investigation exist as hydrazo tautomers in both phases, while dye **7** exists only in the azo form in both solid and solution phases.

Theoretical analysis has been applied to calculate the tautomerization energy, Table 1, for the two tautomers of dyes **3–6** and the azo tautomer of dye **7**, which lacks conjugation to an acidic proton. The geometric optimization of dyes **3–6** revealed that the hydrazo tautomer is more stable, with a lower relative energy (∆E = 24.43—35.72 kcal/mol) than the azo tautomer, which supports the experimental data, see the Supplementary information.

**Table 1:** Energy of tautomers (azo and hydrazo) of dyes **3–7** calculated by DFT using B3LYP/6-31G(d,p) in DMF.

Dye number	Name of tautomer	E (Hartree)	∆E ( kcal/mol)
**3**	Azo	− 1479.678804	27.12
	Hydrazo	− 1479.722016	
**4**	Azo	− 1129.764848	35.72
	Hydrazo	− 1129.821771	
**5a**	Azo	− 1393.412230	32.01
	Hydrazo	− 1393.463249	
**5b**	Azo	− 1855.535928	31.67
	Hydrazo	− 1855.586405	
**6**	Azo	− 1353.637051	24.43
	Hydrazo	− 1353.675976	
**7**	Azo	− 1815.840725	–

### Electronic absorption spectra of azo dyes 3–7

**Figure 3:**
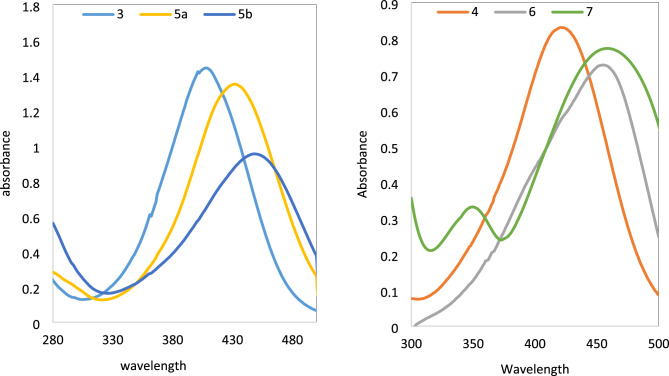
Electronic absorption spectra of bis azo dye **3–7** in DMF.

The experimental and theoretical electronic absorptions data of bis azo dyes **3–7** are compared, Table 2. The B3LYP/6-31G(d,p) method was used to optimize the structures in both tautomeric forms and determine the computed λ_max_ values, which lie in the range of (426–474 nm) for the hydrazo tautomer and in the range of (330–466 nm) for the azo tautomer. In contrast, the experimental absorption of the dyes **3–7** lies in the region (408–462 nm), Table 1 and Fig. 3. The estimated TD-DFT λ_max_ of the hydrazo tautomer for dyes **3–6** agrees with the experimental absorption data.

**Table 2:** The calculated (hydrazo and azo) and experimental absorption maxima (**λ**_**max**_) of bis azo dyes **3–7** in DMF, DMF/0.1 M HCl and DMF/0.1 M NaOH.

Dye no	Observed color	λ_max_ (nm)				
		Experimental DMF	Calculated hydrazone	Calculated Azo	DMF + NaOH	DMF + HCl
**3**	Yellow	408	426	368	432	368
**4**	Yellowish orange	422	444	363	430	404
**5a**	Dark Red	432	464	358	449	418
**5b**	Dark red	448	450	330	456	433
**6**	Yellowish -Orange	455	474	357	506	474
**7**	Slightly Brown	462–350	––	466	467–353	441

The absorption spectra of dyes **3–7** were recorded in DMF. The value of λ_max_ for all dyes depends on the coupler groups with the locality of –NH_2_ (**5–7**) and C = O or enolic hydroxyl group (**3, 5a,b**) group present in the heterocyclic pyrazole moiety. The expected tendency of the higher λ_max_ of dye **6–7** was observed because of the presence of four electron rich NH_2_ groups at the 3,5-positions in the pyrazole rings compared with C = O of dyes **5a,b**. While the higher λ_max_ of dyes **5b** and **7** than those **5a** and **6,** respectively is related to the presence of phenyl groups in the former dyes which increase unsaturation and conjugation.

The first absorption band appeared in the wavelength range 252–260 nm in DMF solution corresponding to the well-known low energy σ–σ* transition. The second band in the rang 408–462 nm is reported for n–π* transition of the hydrazone group^27^. Moreover, absorption band at 332–350 nm attributed to azo structures^28^ is missing for dyes **3–6.** Accordingly, the possibility of existence of azo structures of dyes **3–6**, are rejected and provides support for the hydrazone structure as the most plausible tautomeric structure. While the dye **7** exhibit two absorption band at 462 and 350 nm assigned to the n–π* and π–π* transition corresponding to azo group^29^.

Dyes **5b** and** 7** showed bathochromic shift is presumably due to the presence of extra phenyl rings, While dye **3** exhibited a hypsochromic shift. This suggests that the coupler moieties significantly influence the positions of the absorption bands.

### Effect of acid and base

**Figure 4:**
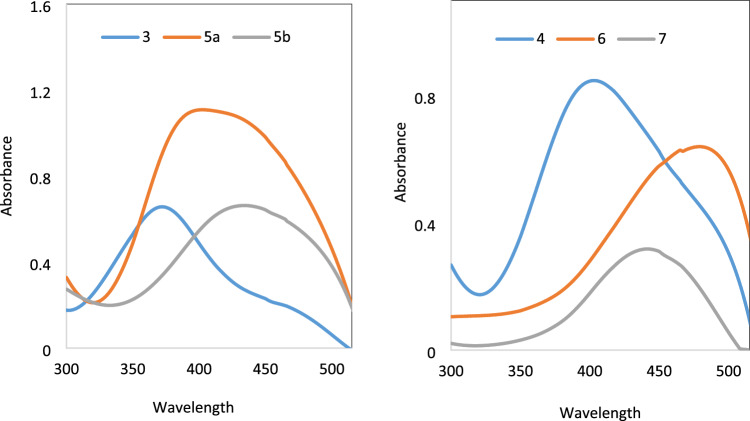
Absorption Spectra of bis azo **3–7** in DMF-H_2_O and 0.1 M HCl.

**Figure 5:**
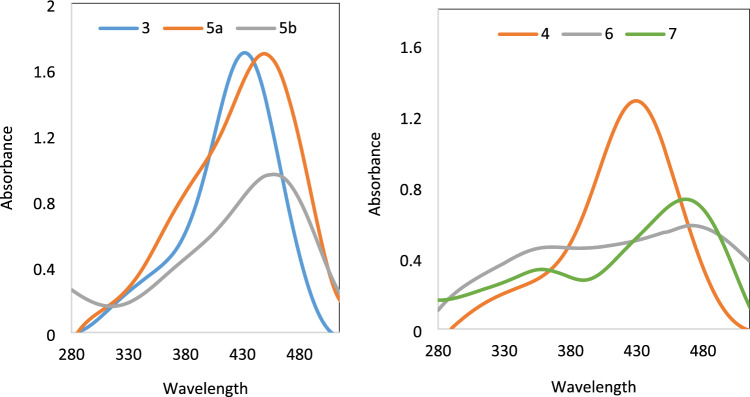
Absorption Spectra of bis azo dyes **3–7** in DMF-H_2_O and 0.1 M NaOH.

Azo dyes are considered as acid–base indicators because they affect the reaction color, which causes a change in UV/vis absorption due to a protonation equilibrium^30^. Due to the presence of four basic nitrogen atoms, the possibility of protonation and deprotonation of bis azo dyes **3–7** in DMF–H_2_O were investigated using 0.1 M sodium hydroxide and 0.1 M hydrochloric acid, Figs. 4 and 5, respectively. The impact of the acid and base on the absorption spectrum of the dye solutions was examined and the results revealed that the absorptions of the titled dyes are quite sensitive to pH medium, Table 2.

It was found that the addition of 0.1 M hydrochloric acid causes a hypsochromic shift in the UV/vis absorption maxima (max) of dyes **3–6** in DMF solution. This is because protonation of hydrazo groups can prevent resonance between biphenyl and nitrogen of hydrazo group. While the addition of acid to dyes **7** in DMF solution showed a small hypsochromic shift of maxima λ_max_, this is because resonance is slightly inhibited due to protonation of azo and/or amino groups.

The slight bathochromic shift of λ_max_ for the bis azo dyes **3–6** upon addition of NaOH in DMF is presumably due to the hydrazo proton is deprotonated. As a result, the conjugation is increased either between anionic form of hydrazo group and pyrazole moiety of dye **4** and** 6** or between anionic form of hydrazo group with cyano group as in dyes** 3** and **4.** On the other hand, dye **7** in DMF solution showed no significant bathochromic shift due to deprotonating process is not freely available and we suggest that dye **7** exists in the azo tautomer in neutral, acid and alkaline media.

### Dyeing process and fastness properties

The disperse insoluble dyes in water that applied from aqueous dispersion rather than from solution was commercially and environmentally developed for coloration of synthetic fibers. The completely insoluble dyes in water were turned application-ready by being dispersed to microscopic-fine particles of the order of a few microns in the presence of dispersing agents. The more hydrophobic fiber could then be dyed using the resulting easily dispersible solid, which partitioned into the fiber from low concentrations of dye bath.

**Figure 6:**
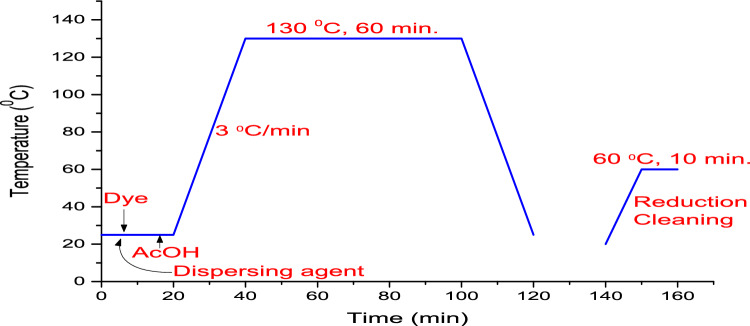
Dyeing method for polyester fibres with dyes **3–7** in water and dispersing agent.

The synthesized disperse dyes under study **3–7** were applied to polyester textiles (PE-F) using a high temperature (HT) dyeing process with a material to liquor ratio of 1:20 at 130 °C. In dyeing process, 2% dye based on the weight of the PE-F was utilized. To enhance the dye solubility in water, DYEWELL-002 was used as a dispersing agent in the dye bath. The dispersed dye was first adsorbed by the surface of PE-F followed by its diffusion through the fiber. The dyeing process was running using aqueous acetic acid at pH 4–5, and then temperature of the dye bath was raised to 130 °C in a dyeing machine under pressure at a rate of 3 °C/min. After being pierced for 60 min, the temperature was cooled to 50 °C. After the dyeing process was finished, the PE-F was rinsed and treated with aqueous solutions of sodium hydrosulphite (1 g/L) and NaOH (1 g/L) to achieve surface reduction, then the dyed PE-F washed with H_2_O and finally was air dried, Fig. 6.

Bis azo dyes **3–7** have been subjected to the standard procedure^31^ for color fastness, which includes washing, hot pressing (scorch) (cotton and polyester), perspiration (acidic and alkaline) and light fastness, Table 3. Gray scale ratings ranging from 1 (poor) to 5 (excellent) were used to evaluate all of the measured properties^32^.

**Table 3:** Fastness properties of bis azo azo disperse dyes **3–7.**

Dyed polyester	Dye no	Wash* fastness	Perspiration fastness(cotton)**		Perspiration fastness (poly ester)		Scorch fastness*** (180 °C)		Light**** fastness
			acidic	alkaline	acidic	alkaline	Cotton	poly ester	
	**3**	5	5	5	5	5	5	5	4–5
	**4**	5	5	5	5	5	4	5	5
	**5a**	5	5	5	5	5	4	5	4
	**5b**	2–3	2–3	3–4	3–4	3–4	4	3	3
	**6**	5	5	5	5	5	4	5	5
	**7**	5	5	5	5	5	5	5	5

*AATCC16 (ISO 105 C06); **AATCC15 (ISO 105 E04); ***AATCC13 (ISO 105 X11); ****AATCC 16 (ISO 105 B02).

Table 3 demonstrates that bis azo dyes **3–7** exhibited excellent fastness levels to washing, perspiration, and scorch on polyester fabric except dye **5b** has moderate fastness levels. In terms of light fastness, dyes **4, 6,** and** 7** displayed good results.

### Dye exhaustion, reflectance and color strength

The exhaustion percentage (%E) of the disperse dyes **3–7** by PE-F in H_2_O was optically calculated by measuring the concentration of dye bath before (C_1_) and after (C_2_) dyeing spectrophotometerically via UV/visible instrument (pg. T80 +) at λ_max_ of the appropriate dye^33^ by Eq. (1)^34^. Dyes **3** and** 5b** demonstrated comparatively excellent %E (above 80%), dyes **4** and** 7** with good %E (above 70%) while dye **5a** and** 6** displayed relatively low %E for PE-F %E (above 60%), Table 4**.**

1 $$\% E = \frac{{C_{1} - C_{2} }}{{C_{1} }}*100$$

**Table 4:** Reflectance (%), colour strength (K/S) value at λ_max_, K/S_sum_ values and Dye exhaustion (E%) of dyed polyester fabrics of bis azo dyes **3–7.**

	Reflectance (%)	K/S	K/S_sum_	Dye exhaustion
3	3.45	13.50	573.7	85.4
4	13.08	2.90	119.2	72.5
5a	27.98	0.93	60.5	65.9
5b	3.98	11.60	777.6	85.2
6	22.39	1.30	86.8	68.9
7	13.67	2.75	169.8	71.8

The quantity of visible light that a color of dyed fabric could reflect was represented by its reflectance value. As a result, white colored dyed fabric reflects all light components and has a 100% light reflectance, whereas black color has 0% reflectance since it absorbs all light components. Therefore, all other colors' reflectance ratings fall between these two extremes. The measured reflectance values of dyed fabric by azo dyes **3–7**, Table 4, were obtained using a UV/vis/NIR-spectrometer Jasco-V-570 (1), across the range of 190–2500 nm. The color strength (K/S ) of the dyed PE-F was determined at λ_max_ by the use of Kubelka–Munk^34^Eq. (2) and given in Table 4.

2 $$K/S = (1 - R)^{2} /2R$$

where K the absorption coefficient, S the scattering coefficient and R is the decimal fraction of the reflection of the dyed fabric.

**Figure 7:**
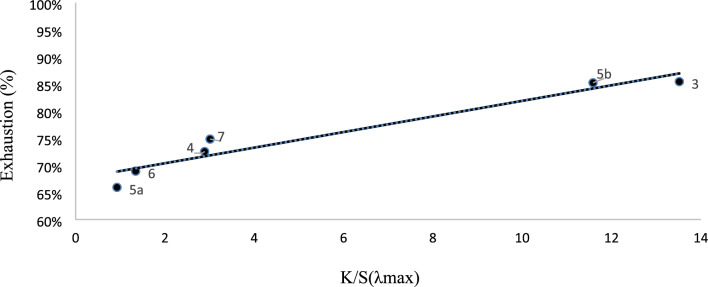
Plots of E% against K/S of dyes **3–7** samples.

Clearly, the current reflectance curves follow a similar pattern with a tiny band shift. As a result, all samples are yellowish orange bag-reddish orange and brown bag. The color strengths (K/S) of dyed PE-F, Table 4, ranged from 0.93 to 13.5, indicating that the coupler moiety mostly determines the color strength. This is obviously observed form the change in color from bright yellow to brown tones when the materials were dyed with dyes **3–7**. While dye **5a** displayed the lowest color strength value, the PE-F sample dyed with dyes **3** and **5b** displayed a high K/S value. There are good agreements between the E% with color strength for dyeing of PE-F by dyes **3–7**, Fig. 7.

The color strength summation (K/Ssum) values are calculated by Eq. (3) for dyes **3–7** samples in the visible spectrum ranging from 390 to 700 nm.

3 $$K/S_{{{\text{sum}}}} = \mathop \sum \limits_{390}^{700} \left( {{\text{K}}/{\text{S}}} \right)_{\lambda }$$

The K/Ssum metric is a widely used measure in the textile industry for evaluating color strength and is an important parameter for assessing the suitability of different dye-coupler combinations for various applications. As shown in Table 4, we observed a clear positive relationship between the K/Ssum value and the color yield for each dye-coupler combination. It was found that dye **5b** produced the darkest brown hue when dyeing PE-F, as evidenced by its highest K/Ssum value among all the dyes tested. On the other hand, dye **5a** produced the lightest color depth, with the lowest K/Ssum value.

Our results also demonstrate that even small differences in the structure of the dyes can have a significant impact on the K/Ssum value and ultimately the color yield. This underscores the importance of carefully selecting the appropriate dye-coupler combination for a given application, taking into account factors such as the fiber type, dyeing conditions, and desired color outcome. In addition to its value in laboratory-scale experiments, the K/Ssum metric has practical applications in industrial settings, where it can be used to assess the color strength and suitability of different dye formulations for large-scale production.

### DFT investigation and dyeing mechanism

Based on its theoretical basis, the density function theory (DFT) is the simplest method for investigating the molecular geometry^19^ and it can be used to postulate the dyeing mechanism according to the dye class. Because of dye bath is acidic (pH 4–5 by acetic acid), theoretical investigation will perform on protonated dye species.

The chemical descriptor parameters, Table 5,^35,36^ of the protonated tautomer forms of the bis azo dyes **3–7** were calculated using the DFT method in order to study dyeing efficiency and to forecast the mechanism of the dyeing process.

**Table 5:** Chemical descriptor parameters of protonated dyes **3–7** in aqueous phase.

Compound Parameter	Dyes					
	**3**	**4**	**5a**	**5b**	**6**	**7**
E_HOMO_	− 0.2198	− 0.2318	− 0.1992	− 0.1985	− 0.2030	− 0.1851
E_LUMO_	− 0.0975	− 0.1118	− 0.0877	− 0.0938	− 0.0898	− 0.0728
∆E	0.1223	0.1200	0.1116	0.1047	0.1132	0.1124
IP	0.2198	0.2318	0.1992	0.1985	0.2029	0.1851
EA	0.0975	0.1118	0.0877	0.0938	0.0898	0.0728
χ	0.1586	0.1718	0.1435	0.1462	0.1464	0.1290
µ eV	− 0.1586	− 0.1718	− 0.1435	− 0.1462	− 0.1464	− 0.1290
η	0.0611	0.0601	0.0558	0.0523	0.0566	0.0562
σ	16.3586	16.6612	17.9259	19.1113	17.6741	17.8016
ω	0.2057	0.2457	0.1844	0.2041	0.1893	0.1480
µ (D)	5.3932	4.2716	2.5998	4.4236	2.5831	3.7215

The ionisation potential IP and electron affinities EA values of any chemical species have been related to their lowest E_LUMO_ and highest occupied molecular orbitals E_HOMO_, respectively^35,36^, Eqs. (4–5).

4 $${\text{IP}} = - {\text{E}}_{{{\text{HOMO}}}}$$

5 $${\text{EA}} = - {\text{E}}_{{{\text{LUMO}}}}$$

Energy gap (ΔE)^37^, chemical potential (μ), absolute electronegativity (χ), absolute softness (σ) and absolute hardness (η) global parameters are calculated using Eq. (6–10) ^38^.

6 $$\Delta {\text{E}} = {\text{E}}_{{{\text{LUMO}}}} - {\text{E}}_{{{\text{HOMO}}}}$$

7 $${\upchi } = \frac{{{\text{IP}} + {\text{EA}}}}{2}$$

8 $$\mu = - \chi$$

9 $$\eta = \frac{IP - EA}{2}$$

10 $$\sigma = \frac{1}{\eta }$$

The energy lowering caused by the maximum electron flow between the donor and acceptor was measured by the electrophilicity index parameter ω^39^, Eq. (11).

11 $$\omega = \frac{{\mu^{2} }}{2\eta }$$

The inclination of a molecule to transfer electrons to acceptor molecules with lower energy MO is reflected by the high values of the E_HOMO_. The energy E_LUMO_ represents a molecule's capacity to take electrons^40^. In general, as the HOMO and LUMO energy levels of a molecule change, so does its capacity to bind. In other words, independent of lowering HOMO and increasing LUMO energy levels, a molecule's capacity to bind is improved when ∆E value is lowered. i.e. Accordingly, the dyeing strength increases when the dye molecule has a low ∆E value. While the softness parameter σ will reflect strong dyeing strength, the greater values of chemical hardness η will provide low dyeing for fibre.

The electrophilicity index ω^41^ is a description that might depict the dye power of chemical species. The molecule's global electrophilicity index enables quantitative assessment of its reactivity^42–44^. The electrophilicity index displays the ability of the electron-accepting ability^45^. It should be emphasised that strong colour strength should have low electronegativity values^46^. As a result, the increase in chemical potential causes increasing of dyeing strength.

Table 5 points out that, dye **3** has the highest dyeing strength based on parameters E_HOMO_, E_LUMO_, *∆E, η* and* S* while dye **4** is the highest depending on parameters **χ** and ***µ* eV.** The correlation results of each calculated parameter with %E and K/S revealed that color strength (K/S) and %E depend mainly on the effect of dipole moment. The higher in dipole moment in dyes **3** and **4** (5.3932 and 4.2716, respectively), the higher in both K/S and %E. The cyclized pyrazole dyes **5a,b** and **6–7** showed lower in dipole moment than their parent dyes **3** and **4** which correlate with their lower value of K/S and %E, respectively.

#### Dyeing mechanism

**Figure 8:**
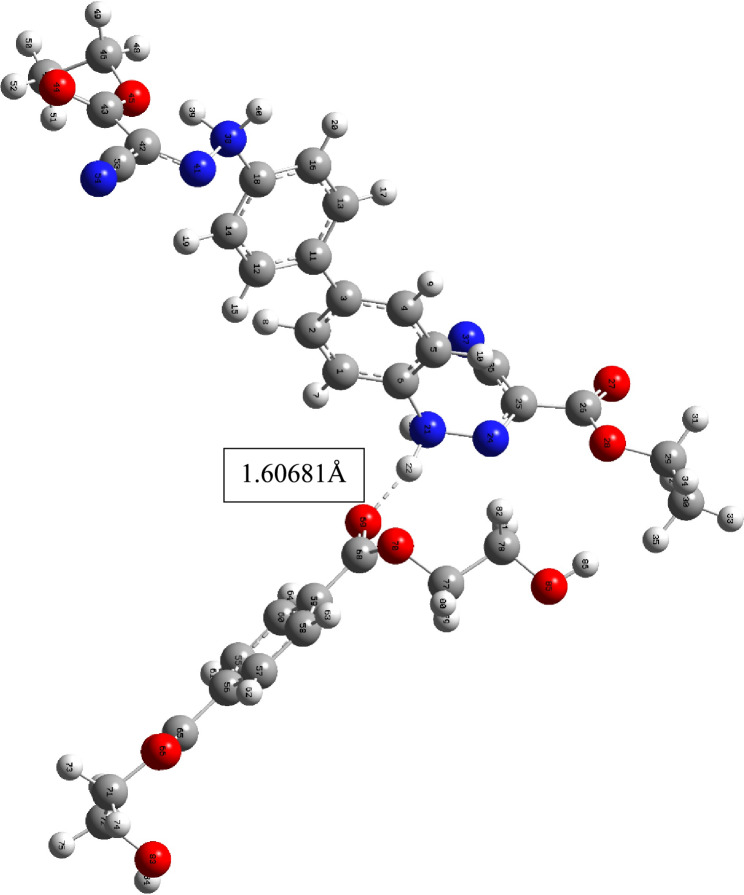
The optimized structure of dye **3** and methyl terephthalate monomer and the bond lengths between the hydrazo hydrogen atom with the carbonyl oxygen of the methyl terephthalate monomer.

**Figure 9:**
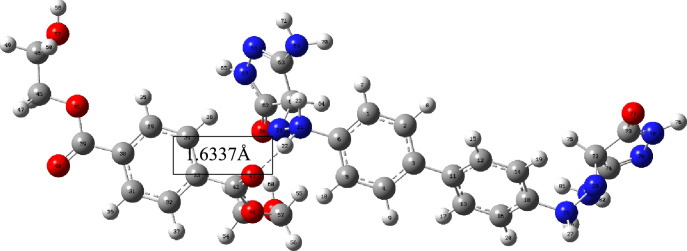
The optimized structure of dye **5a** and methyl terephthalate monomer and the bond lengths between the hydrazo hydrogen atom with the carbonyl oxygen of the methyl terephthalate monomer.

The proposed method for dyeing using protonated tautomers of dyes **3–7** involves the initial adsorption of the dye onto the PE surface via hydrogen bonding and/or electrostatic attraction. The polyester's carbonyl oxygen and/or etheric linkages can create a hydrogen bond with the protonated hydrazo moiety from the dye (NH….O = C) with length = 1.60681 Å for dye **3** and length = 1.6337 Å for dye **5a**. Finally, the diffusion process is completed by heating, Figs. 8 and 9.

## Conclusion

The bis azo dyes **3–6** were found to exist in the hydrazone tautomer in both the solid and solution states, while dye **7** existed in the azo form due to the lack of a conjugation to an acidic proton. The estimated TD-DFT λ_max_ of the titled dyes agreed with the experimental absorption data. Dyes **5b** and **7** showed bathochromic shift, whereas dye **3** exhibited a hypsochromic shift, suggesting that the coupler moieties significantly influence the positions of the absorption bands. The absorption spectra of the titled dyes were found to be highly sensitive to the pH medium, as demonstrated by the results of acid and base addition. The change in the coupler moieties affected the color shading of dyed polyester fabrics. Among the disperse dyes, fabric dyed with dye **5a** (K/S = 11.60) had the deepest shade, while fabric dyed with dye **5b** (K/S = 0.93) had the lightest shade. Dyes **3–7** exhibited excellent fastness levels to washing, perspiration, and scorching on polyester fabric, except dye **5b**, which exhibited moderate fastness levels. Dyes **3** and **5b** displayed excellent %E, while dyes **4** and **7** have good %E (above 70%). The correlation analysis between the calculated chemical descriptor and the dyeing data (%E and K/S) indicated that K/S and %E mainly dependent on the dipole moment.

## Experimental

### General method for synthesis of dyes 3, 4

#### Step 1: Preparation of diazonium 2

Benzidine **1** (5.52 g, 0.03 mol) was placed in a 250 mL conical flask, a mixture of 15 mL conc. HC1 with 10 mL water was added and stirring till clear solution, the mixture kept at 0 °C in an ice bath. Mixture of sodium nitrite (4.14 g, 0.06 mol) in 10 mL cold water added to the benzidine hydrochloride mixture slowly with constant stirring.

#### Step 2: Reaction of diazonium salt with coupling reagent

The former prepared diazonium salt solution **2** was then added dropwise to the coupler solution, namely, ethyl cyanoacetate and malononitrile, (0.06 mol) in 17 mL ethanol and 30 g sodium acetate in 15 mL of water (keep the mixture alkaline) at 0 °C. The progress of the reaction was monitored by TLC and then crude dyes were filtered, washed with hot water for several times.

### General method for synthesis of compounds 5a, and 6

A mixture of compound **3** or **4** (0.004 mol) and hydrazine hydrate 98% (0.008 mol) was refluxed in 15 mL ethanol for 4 h. The progress of the reaction was monitored by TLC. The formed precipitate was filtered, dried and recrystallized from methylene chloride.

### General method for synthesis of compounds 5b, and 7

A mixture of compound **3** or **4** (0.004 mol) and phenyl hydrazine (0.008 mol) was refluxed in 15 mL ethanol for 4 h. The progress of the reaction was monitored by TLC. The formed precipitate was filtered, dried and recrystallized from methylene chloride.

#### Diethyl 2,2'-([1,1'-biphenyl]-4,4'-diylbis(hydrazin-2-yl-1-ylidene))bis(2-cyanoacetate) 3

Yellow powder, 9.9 g (85%) yield; m.p.210–212 ºC. UV: ג_max_ (DMF) 408 nm, and Ɛ_max_ 4166 mol^− 1^dm^3^cm^− 1^. IR (KBr): ῡ 3217 (N–H), 3047 (C–H), 2210 (C≡N), 1733 (C = O), and 1597(C = N) cm^− 1^. ^1^H NMR (DMSO-*d6*, 500 MHz): δ 12.36 (2H, s, 2NH), 7.75 (4H, d, *J* = 8.3 Hz, 4Ar-H), 7.57 (4H, d, *J* = 8.3 Hz, 4Ar-H), 4.31 (4H, q, *J* = 6.7 Hz, 2CH_2_), 1.32 (6H, t, *J* = 7.2 Hz, 2CH_3_) ppm. ^13^C NMR (APT) (DMSO-*d6*, 125 MHz): δ 161.0, 141.2, 135.7, 127.3, 116.6, 111.6, 103.8, 61.6, and 14.2 ppm. C_22_H_20_N_6_O_4_ requires: C, 61.09; H, 4.67; N, 19.44% found: C, 61.51; H, 4.52; N, 19.42%

#### *N*',*N*''-([1,1'-biphenyl]-4,4'-diyl)dicarbonohydrazonoyl dicyanide 4

Yellowish orange powder, 8.6 g (94%) yield; m.p. above 300 ºC. UV: ג_max_ (DMF) 422 nm, and Ɛ_max_ 2766 mol^− 1^dm^3^cm^− 1^. IR (KBr): ῡ 3414 (N–H), 3064 (C–H sp2), 2220 (C≡N), and 1605(C = N) cm^− 1^. ^1^H NMR (DMSO-*d6*, 500 MHz): δ 13.13 (2H, s, 2NH), 7.73 (4H, d, J = 8.2 Hz, 4Ar-H), 7.54 (4H, d, *J* = 8.1 Hz, 4Ar-H). ^13^C NMR (APT) (DMSO-*d6*, 125 MHz): δ 141.4, 136.8, 127.9, 117.5, 114.9, 110.6, and 85.1 ppm. C_18_H_10_N_8_ requires: C, 78.10; H, 3.00; N, 33.12% found: C, 78.41; H, 3.17; N, 33.42%

#### 4,4'-([1,1'-biphenyl]-4,4'-diylbis(hydrazin-2-yl-1-ylidene))bis(5-amino-2,4-dihydro-3*H*-pyrazol-3-one) 5a

Yellowish-Orange powder, 1.6 g (85%) yield; m.p. above 300 ºC. UV: ג_max_ (DMF) 432 nm, and Ɛ_max_ 4500 mol^− 1^dm^3^cm^− 1^. IR (KBr): ῡ 3366 (NH_2_), 3182 (N–H), 3040 (C–H sp^2^), 1636 (C = O), and 1592 (C = N) cm^− 1^. ^1^H NMR (DMSO-*d6*, 500 MHz): δ 12.99 (2H, s, 2NH), 10.55 (2H, s, 2NH), 7.73 (4H, d, *J* = 8.7 Hz, 4Ar-H), 7.61 (4H, d, *J* = 8.6 Hz, 4Ar-H), 5.88 (4H, s, 2 NH_2_). ^13^C NMR (APT) (DMSO-*d6*, 125 MHz): δ 123.5, 114.7, 99.8, 92.6, 80.5, 79.2, and 10.1 ppm. C_18_H_16_O_2_N_10_ requires: C, 53.45; H, 3.99; N, 34.64; found: C, 53.24; H, 3.87; N, 34.52%

#### 4,4'-([1,1'-biphenyl]-4,4'-diylbis(hydrazin-2-yl-1-ylidene))bis(5-amino-2-phenyl-2,4-dihydro-3H-pyrazol-3-one) 5b

Dark brown powder, 2.14 g (83%) yield; m.p. above 300 ºC. UV: ג_max_ (DMF) 448 nm, and Ɛ_max_ 3180 mol^− 1^dm^3^cm^− 1^. IR (KBr): ῡ 3419 (NH_2_), 3227 (NH), 3010 (C–H sp^2^), 1623 (C = O), and 1562 (C = N) cm^− 1^. ^1^H NMR (DMSO-*d6*, 500 MHz): δ 13.01 (2H, s, 2NH), 7.92 (4H ,d, *J* = 8.0 Hz, 4Ar-H), 7.77 (4H, d, *J* = 8.5 Hz, 4Ar-H), 7.73 (4H, d, *J* = 8.4 Hz, 4Ar-H), 7.39 (4H, t, *J* = 7.8 Hz, 4Ar-H), 7.11 (2H, t, *J* = 7.3 Hz, 2Ar-H) 6.46 (4H, s, 2 NH_2_). ^13^C NMR (APT) (DMSO-*d6*, 125 MHz): δ 156.3, 151.7, 139.5, 136.6, 129.8, 128.2, 124.7, 123.9, 122.3, 118.1, and 117.2 ppm. C_30_H_24_O_2_N_10_ requires: C, 64.73; H, 4.35; N, 25.16; found: C, 64.52; H, 4.27; N, 25.42%

#### 4,4'-([1,1'-biphenyl]-4,4'-diylbis(hydrazin-2-yl-1-ylidene))bis(4*H*-pyrazole-3,5-diamine) 6

Slightly Brown powder, 2.2 g (93%) yield; m.p. above 300 ºC. UV: ג_max_ (DMF) 455 nm, and Ɛ_max_ 5233 mol^− 1^dm^3^cm^− 1^. IR (KBr): ῡ 3397 (NH_2_), 3195 (N–H), 3040 (C–H sp^2^), and 1617(C = N) cm^− 1^. ^1^H NMR (DMSO-*d6*, 500 MHz): δ 10.79 (2H, s, 2NH), 7.77 (8H, s, 8Ar-H), 6.17 (8H, s, 4 NH_2_). ^13^C NMR (APT) (DMSO-*d6*, 125 MHz): δ 153.2, 151.7, 138.1, 127.2, 121.5, and 115.0 ppm. C_18_H_18_N_12_ requires: C, 53.71; H, 4.52; N, 41.77% found: C, 54.01; H, 4.27; N, 41.42%

#### 4,4'-([1,1'-biphenyl]-4,4'-diylbis(diazene-2,1-diyl))bis(1-phenyl-1*H*-pyrazole-3,5-diamine) 7

Slightly Brown powder, 3.14 g (96%) yield; m.p. above 300 ºC. UV: ג_max_ (DMF) 458 nm, and Ɛ_max_ 2450 mol^− 1^dm^3^cm^− 1^. IR (KBr): ῡ 3340 (NH_2_), 3056 (C–H sp^2^), 1625 (C = N), and 1552 (N = N) cm^− 1^. ^1^H NMR (DMSO-*d6*, 500 MHz): δ 7.86 (4H ,d, *J* = 8.1 Hz, 4Ar-H), 7.80 (4H, d, *J* = 8.6 Hz, 4Ar-H), 7.58 (4H, d, *J* = 7.9 Hz, 4Ar-H), 7.49 (4H, t, *J* = 7.4 Hz, 4Ar-H), 7.29 (2H, t, *J* = 7.3 Hz, 2Ar-H) 6.75 (4H, s, 2 NH_2_), 6.23 (4H, s, 2 NH_2_). ^13^C NMR (APT) (DMSO-*d6*, 125 MHz): δ 163.2, 152.5, 150.6, 149.1, 138.5, 127.8, 125.9, 122.5 and 117.8 ppm. C_30_H_26_N_12_ requires: C, 64.95; H, 4.73; N, 30.31% found: C, 64.51; H, 4.57; N, 30.42%

## Supplementary Information

Supplementary Information.

## Data Availability

The datasets used and/or analysed during the current study available from the corresponding author on reasonable request.
