# Supplementary material for: Synthesis, spectroscopic characterization and dyeing performance of novel bis azo dyes derived from benzidine
Source: Sci Rep. 2023 May 15;13:7826. doi: 10.1038/s41598-023-34660-4 (PMC10185538; doi:10.1038/s41598-023-34660-4)
Supplement: Supplementary file 1 — Supplementary Information. [file 41598_2023_34660_MOESM1_ESM.docx]

**Synthesis, Spectroscopic Characterization and Dyeing Performance of Novel Bis Azo Dyes Derived From Benzidine**

Alaa Z. Omar*^a^, Mohamed A. El-Rahman^a^, Ezzat A. Hamed^a^, Samir K. El-Sadany^a^ and Mohamed A. El-atawy^a,b^

^a^ Chemistry Department, Faculty of Science, Alexandria University, P.O. 426 Ibrahemia, Alexandria 21321, Egypt

^b^ Chemistry Department, Faculty of Science, Taibah University, Yanbu 46423 Saudi Arabia.

^*^ Correspondence: Alaazaki@alexu.edu.eg; Tel.: +201111361784, P.O. 426 Ibrahemia, Alexandria 21321, Egypt

Instruments and Apparatus:

Melting points were determined by MEL-TEMP II melting point apparatus in open glass capillaries. The IR spectra were recorded as potassium bromide (KBr) discs on a Perkin-Elemer FT-IR (Fourier-Transform Infrared Spectroscopy), Faculty of Science, Alexandria University. The NMR spectra were carried out at ambient temperature (~25 ºC) on a (JEOL) 500 MHz spectrophotometer using tetramethylsilane (TMS) as an internal standard, NMR Unit, Faculty of Science, Mansoura University. Elemental analyses were analyzed at the Regional Center for Mycology and Biotechnology, Al-Azhar University, Cairo, Egypt. All dyeing processes were carried out using a laboratory sample dyer (DL-6000P/S-3) (starlet-3).

**Materials and chemicals**

Benzidine, malononitrile, ethyl cyanocetate, phenyl hydrazine, hydrazine hydrate, sodium hydroxide (NaOH), sodium carbonate (Na_2_CO_3_), concentrated HCl, methanol, ethanol, dimethyl formamide (DMF) and methylene chloride were purchased from Sigma-Aldrich and were used without further purification. For TLC purpose, methanol: ethyl acetate eluent is used, and the purified dyes were dried by standard method.

**Fastness properties measurement**

***Fastness to Washing***

A Launder-O-meter was used to determine color fastness to washing (UK). The specimens (5 x 4cm) were encased in two pieces of cotton and polyester fabric. The composite specimen was immersed in a 50:1 liquor ratio aqueous solution containing 5 g/L non-ionic detergent and 2 g/L sodium carbonate.

The bath temperature was set to 95 °C for 45 minutes. The samples were then removed, rinsed twice at 40 °C for one minute with occasional stirring or hand squeezing, scouring in 100 mL of acetic acid (0.014%) solution for one minute at room temperature, rinsing again in 100 mL water at room temperature, and drying. The staining on the undyed adjacent fabric was evaluated using the following criteria:1-poor, 2-fair, 3-moderate, 4-good, and 5-excellent.

**Fastness to Perspiration (acid or alkaline)**

The samples were produced by stitching pieces of dyed polyester fabric between two similar pieces of cotton fabric or two similar pieces of polyester fabric, all of equal diameter, and then engrossed them in the acid or alkaline medium for 30 minutes. The staining on the undyed adjacent fabric was graded on a five-point scale: 1-poor, 2-fair, 3-moderate, 4-good, and 5-excellent. The acid solution (pH 3.5) contained sodium chloride (10 g/L), lactic acid (1 g/L), disodium orthophosphate (1 g/L), and histidine monohydrochloride (0.25 g/L). The alkaline solution (pH 8) contained 5 g/L sodium chloride, 2.5 g/L disodium orthophosphate, and 0.5 g/L histidine monohydrochloride, and was adjusted to pH 8.0 with 0.1 N sodium hydroxide.

**Fastness to Sublimation (Scorch fastness)**

The AATTC 107-1999 test was used to determine sublimation fastness. A test fabric sample in contact with undyed fabrics cotton or polyester is exposed to dry heat by coming into close contact with a heated medium (180 ^o^C). Any staining or change in tone on the undyed adjacent fabrics was graded on a 5-point scale: 1-poor, 2-fair, 3-moderate, 4-good, and 5-excellent.

***Assessment of Light Fastness***

The dyed polyester was tested for light fastness on a Xenotest 150 [Original Hanau, chamber temperature 25-30 ^o^C, black panel temperature 60 °C, relative humidity 50-60%, and dark glass (UV) filter system] for 40 hours. The color changes were graded using the following blue scale: 1-poor, 3-moderate, 4-good, and 5-very good.


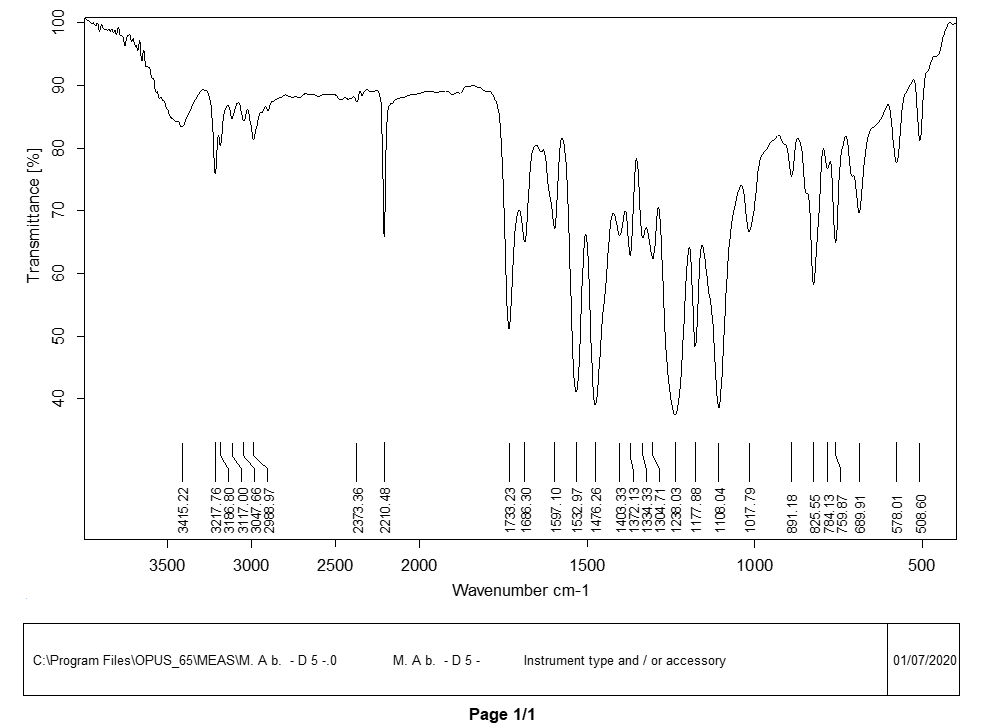


C=O

NH

C≡N

Figure S1. IR (KBr) spectrum of diethyl 2,2'-([1,1'-biphenyl]-4,4'-diylbis(hydrazin-2-yl-1-ylidene))bis(2-cyanoacetate) **3**


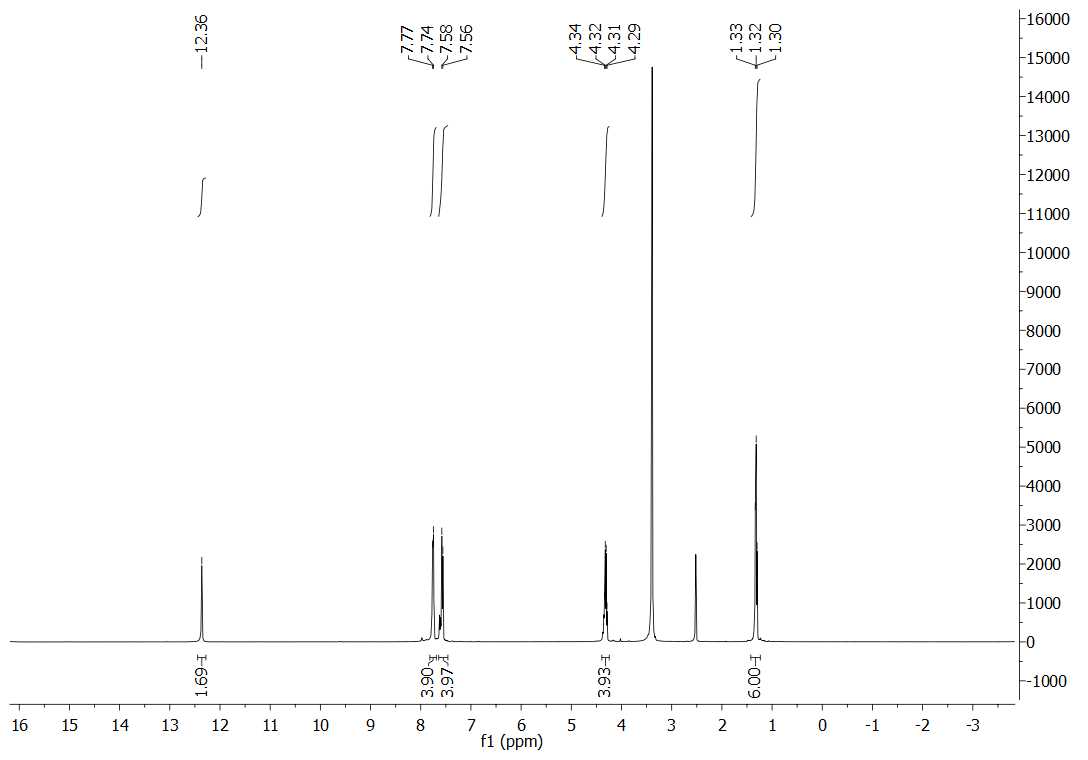


CH_3_

CH_2_

NH

Figure S2.^1^H NMR (DMSO-*d6*, 500 MHz) spectrum of diethyl 2,2'-([1,1'-biphenyl]-4,4'-diylbis(hydrazin-2-yl-1-ylidene))bis(2-cyanoacetate) **3**


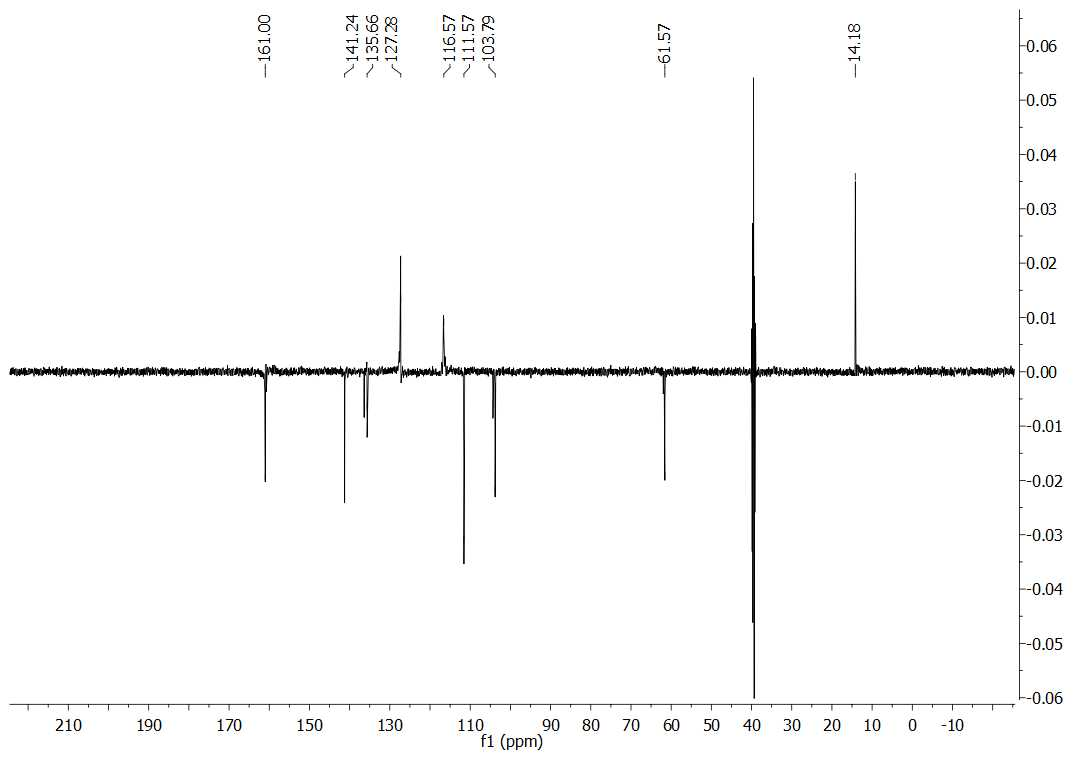


Figure S3.^13^C APT NMR (DMSO-*d6*, 125 MHz) spectrum of diethyl 2,2'-([1,1'-biphenyl]-4,4'-diylbis(hydrazin-2-yl-1-ylidene))bis(2-cyanoacetate) **3**

NH

C≡N

Figure S4.IR (KBr) spectrum of *N*',*N*''-([1,1'-biphenyl]-4,4'-diyl)dicarbonohydrazonoyl dicyanide **4**


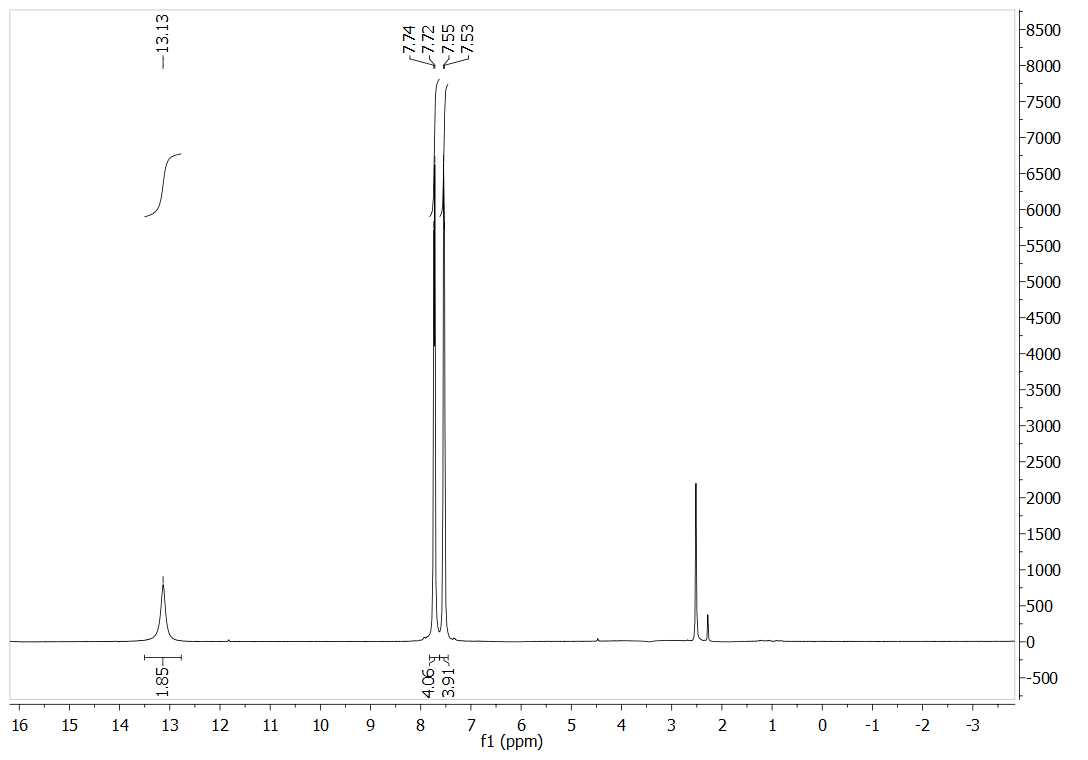


NH

Figure S5.^1^H NMR (DMSO-*d6*, 500 MHz) spectrum of *N*',*N*''-([1,1'-biphenyl]-4,4'-diyl)dicarbonohydrazonoyl dicyanide **4**


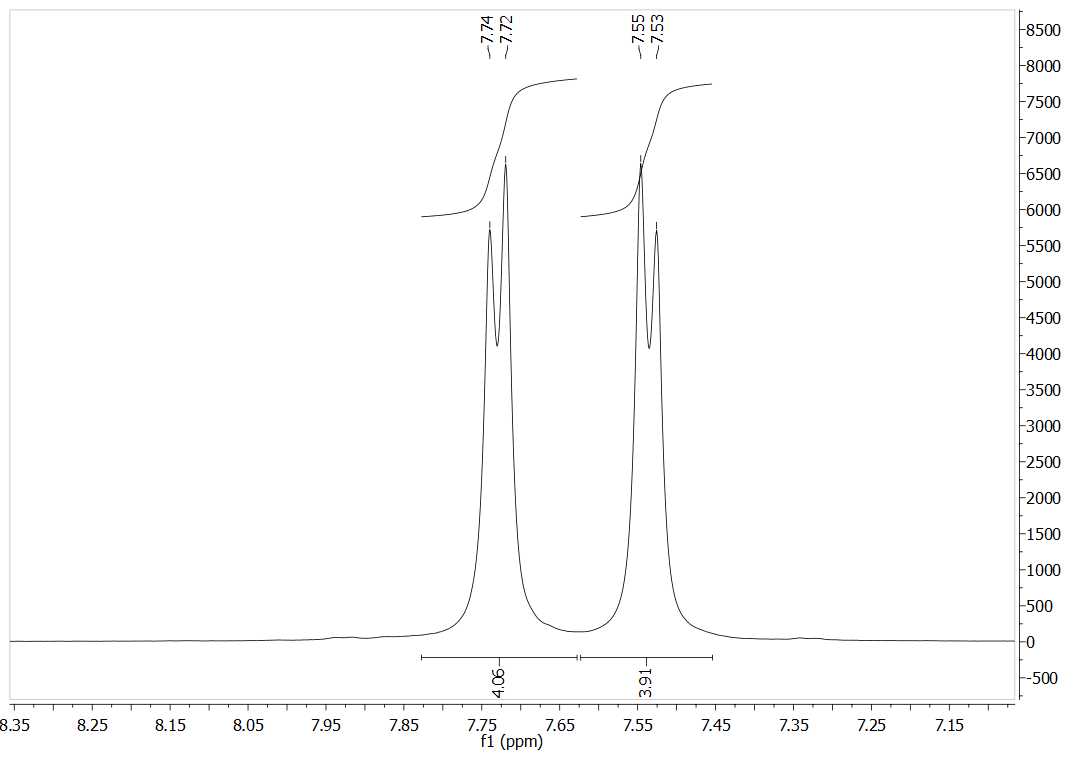


Figure S6.^1^H NMR (DMSO-*d6*, 500 MHz) spectrum of *N*',*N*''-([1,1'-biphenyl]-4,4'-diyl)dicarbonohydrazonoyl dicyanide **4**


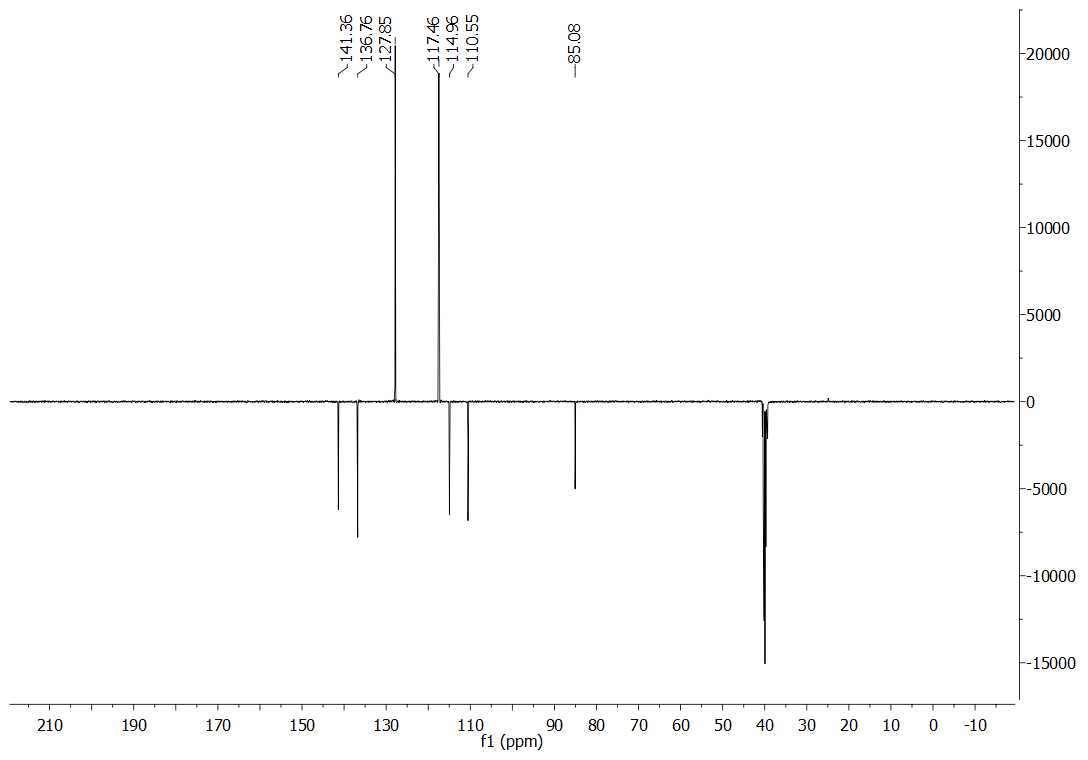


Figure S7.^13^C APT NMR (DMSO-*d6*, 500 MHz) spectrum of *N*',*N*''-([1,1'-biphenyl]-4,4'-diyl)dicarbonohydrazonoyl dicyanide **4**

C=O

NH_2_

NH

Figure S8.IR (KBr) spectrum of 4,4'-([1,1'-biphenyl]-4,4'-diylbis(hydrazin-2-yl-1-ylidene))bis(5-amino-2,4-dihydro-3*H*-pyrazol-3-one) **5a**


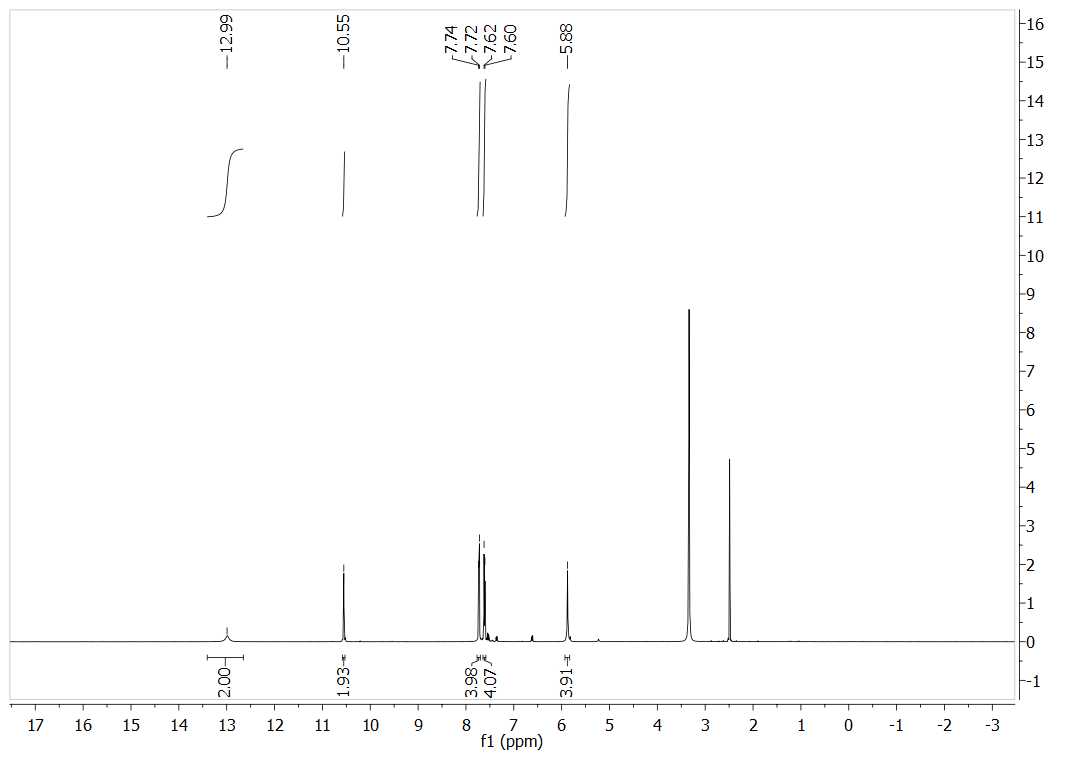


NH_2_

NH

NH

Figure S9.^1^H NMR (DMSO-*d6*, 500 MHz) spectrum of 4,4'-([1,1'-biphenyl]-4,4'-diylbis(hydrazin-2-yl-1-ylidene))bis(5-amino-2,4-dihydro-3*H*-pyrazol-3-one) **5a**


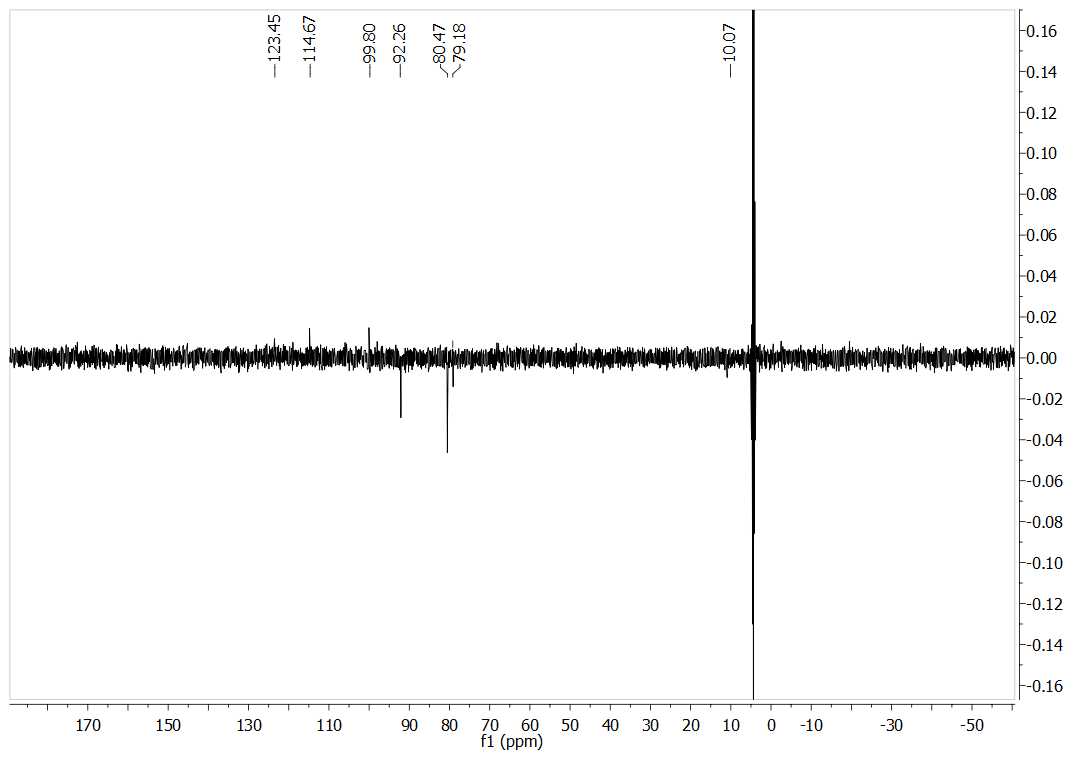


Figure S10.^13^C APT NMR (DMSO-*d6*, 500 MHz) spectrum of 4,4'-([1,1'-biphenyl]-4,4'-diylbis(hydrazin-2-yl-1-ylidene))bis(5-amino-2,4-dihydro-3*H*-pyrazol-3-one) **5a**

NH_2_

C=O

Figure S11.IR (KBr) spectrum of 4,4'-([1,1'-biphenyl]-4,4'-diylbis(hydrazin-2-yl-1-ylidene))bis(5-amino-2-phenyl-2,4-dihydro-3H-pyrazol-3-one) **5b**


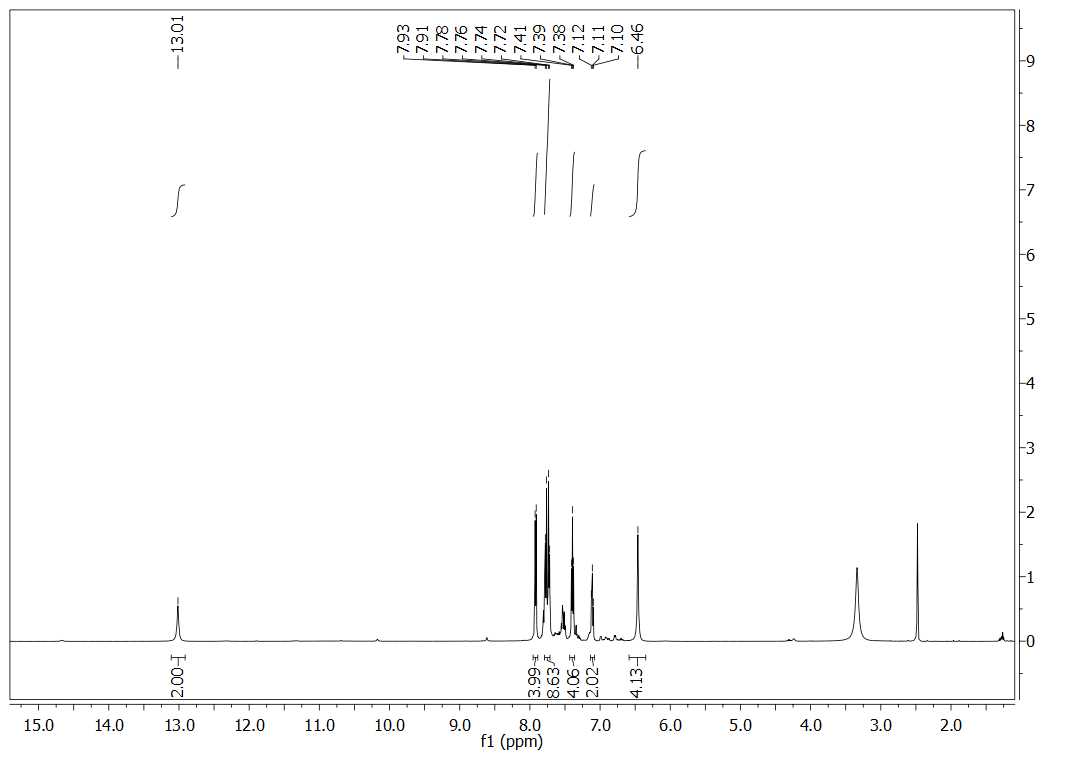


NH

NH_2_

Figure S12.^1^H NMR (DMSO-*d6*, 500 MHz) spectrum of 4,4'-([1,1'-biphenyl]-4,4'-diylbis(hydrazin-2-yl-1-ylidene))bis(5-amino-2-phenyl-2,4-dihydro-3H-pyrazol-3-one) **5b**


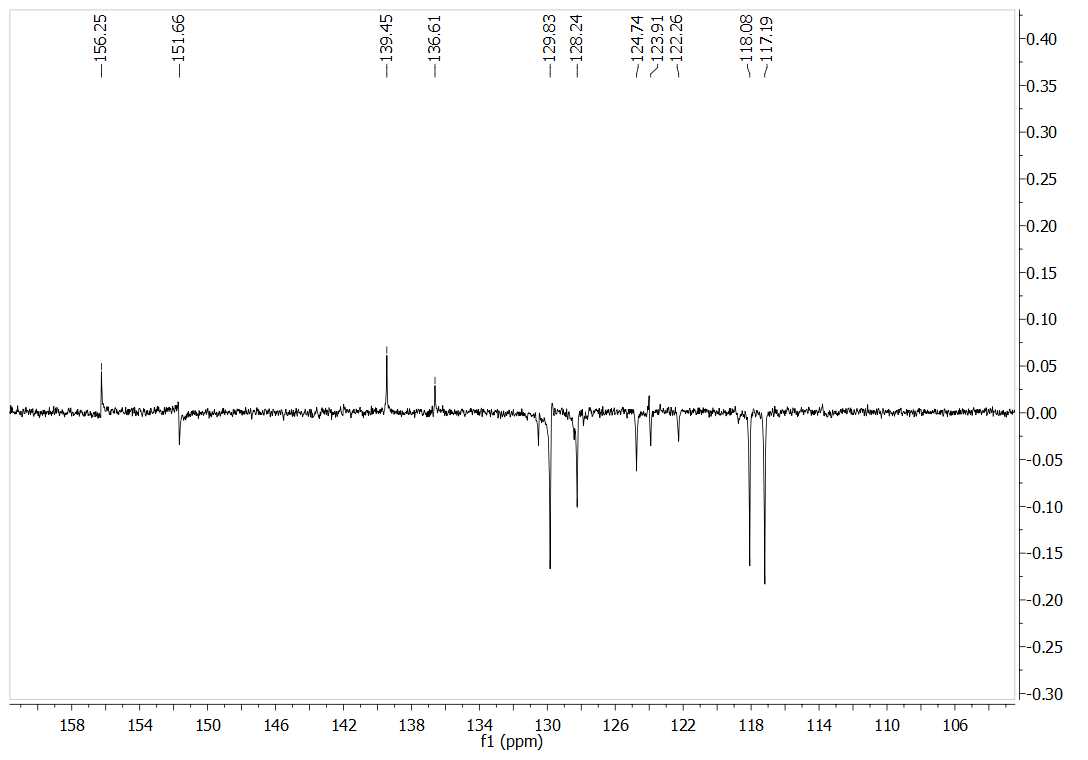


Figure S13.^13^C APT NMR (DMSO-*d6*, 500 MHz) spectrum of 4,4'-([1,1'-biphenyl]-4,4'-diylbis(hydrazin-2-yl-1-ylidene))bis(5-amino-2-phenyl-2,4-dihydro-3H-pyrazol-3-one) **5b**

C=N

NH_2_

Figure S14. IR (KBr) spectrum of 4,4'-([1,1'-biphenyl]-4,4'-diylbis(hydrazin-2-yl-1-ylidene))bis(4*H*-pyrazole-3,5-diamine) **6**

**
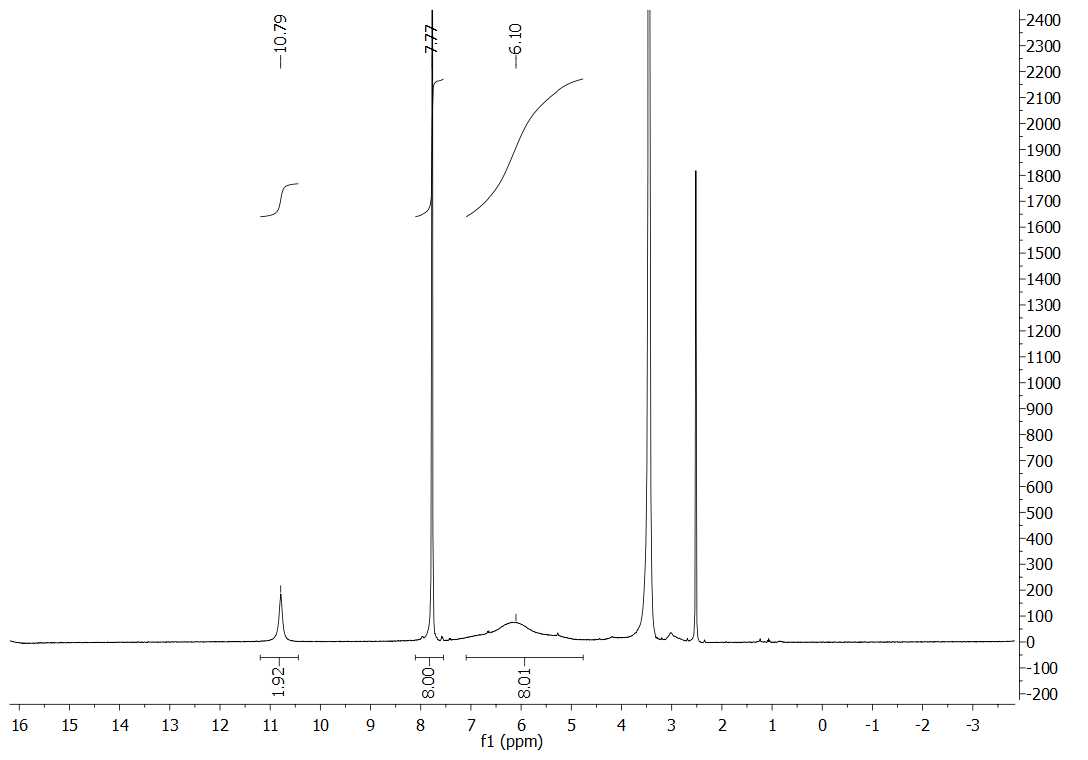
**

NH_2_

NH

Figure S15.^1^H NMR (DMSO-*d6*, 500 MHz) spectrum of 4,4'-([1,1'-biphenyl]-4,4'-diylbis(hydrazin-2-yl-1-ylidene))bis(4*H*-pyrazole-3,5-diamine) **6**


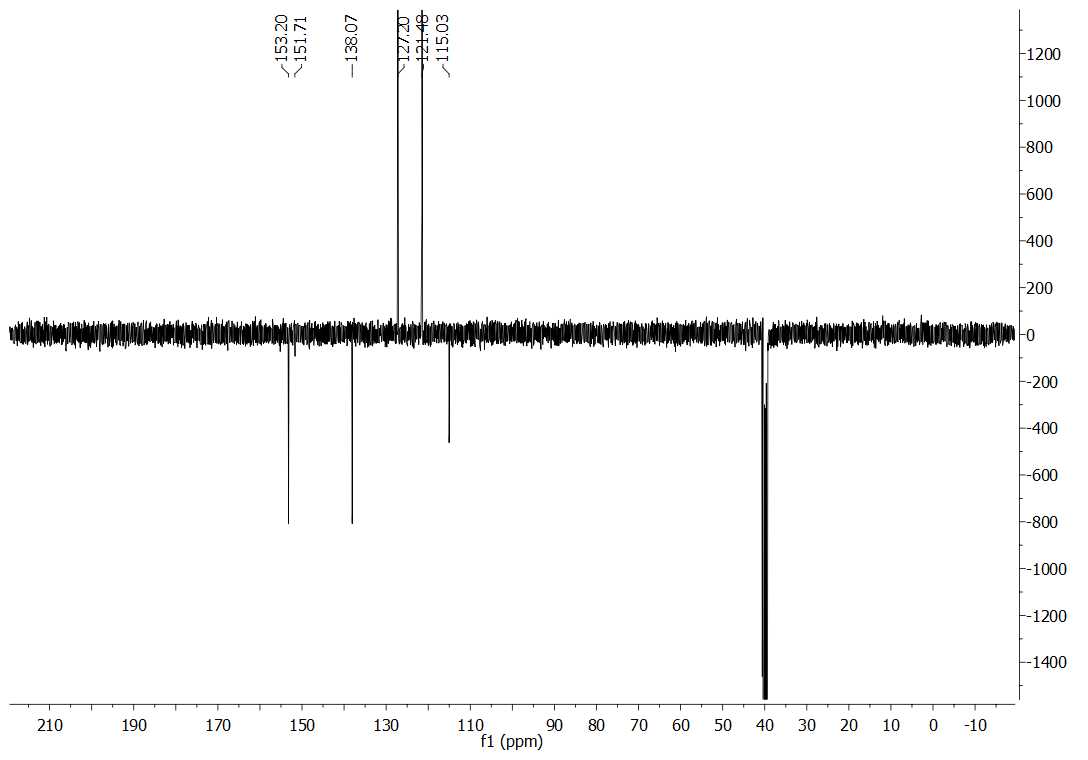


Figure S16.^13^C APT NMR (DMSO-*d6*, 500 MHz) spectrum of 4,4'-([1,1'-biphenyl]-4,4'-diylbis(hydrazin-2-yl-1-ylidene))bis(4*H*-pyrazole-3,5-diamine) **6**

Figure S17. IR (KBr) spectrum of 4,4'-([1,1'-biphenyl]-4,4'-diylbis(diazene-2,1-diyl))bis(1-phenyl-1*H*-pyrazole-3,5-diamine) **7**

N=N

C=N

NH_2_


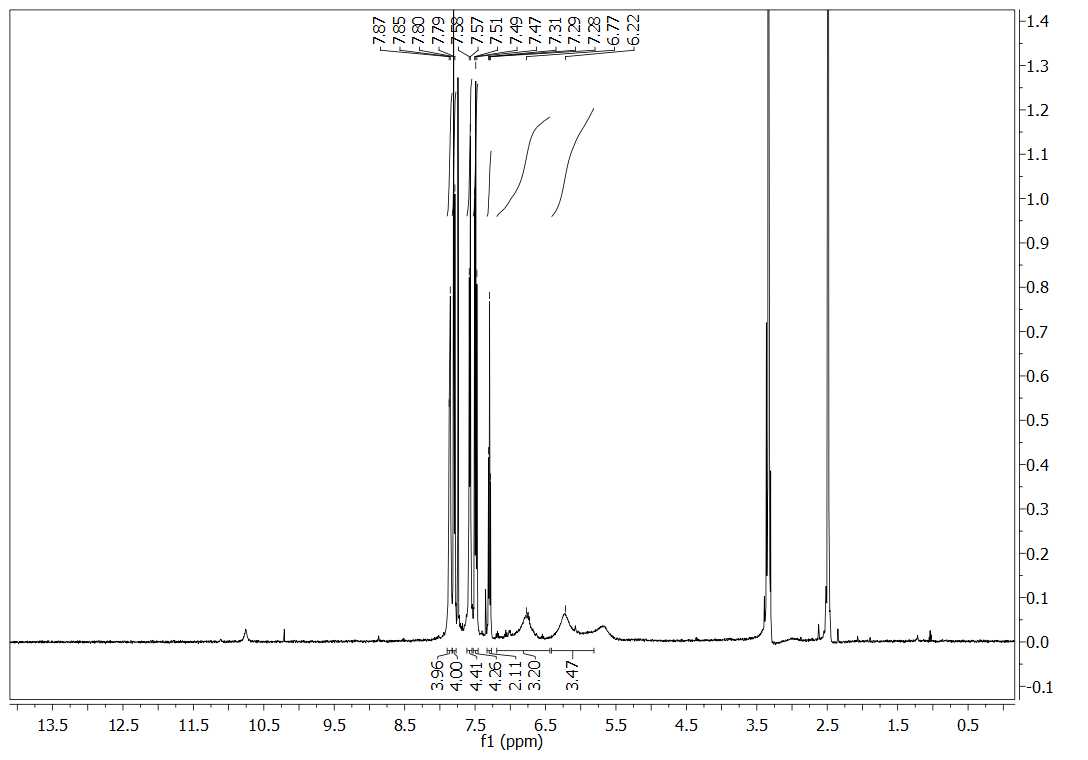


Figure S18. ^1^H NMR (DMSO-*d6*, 500 MHz) spectrum of 4,4'-([1,1'-biphenyl]-4,4'-diylbis(diazene-2,1-diyl))bis(1-phenyl-1*H*-pyrazole-3,5-diamine) **7**


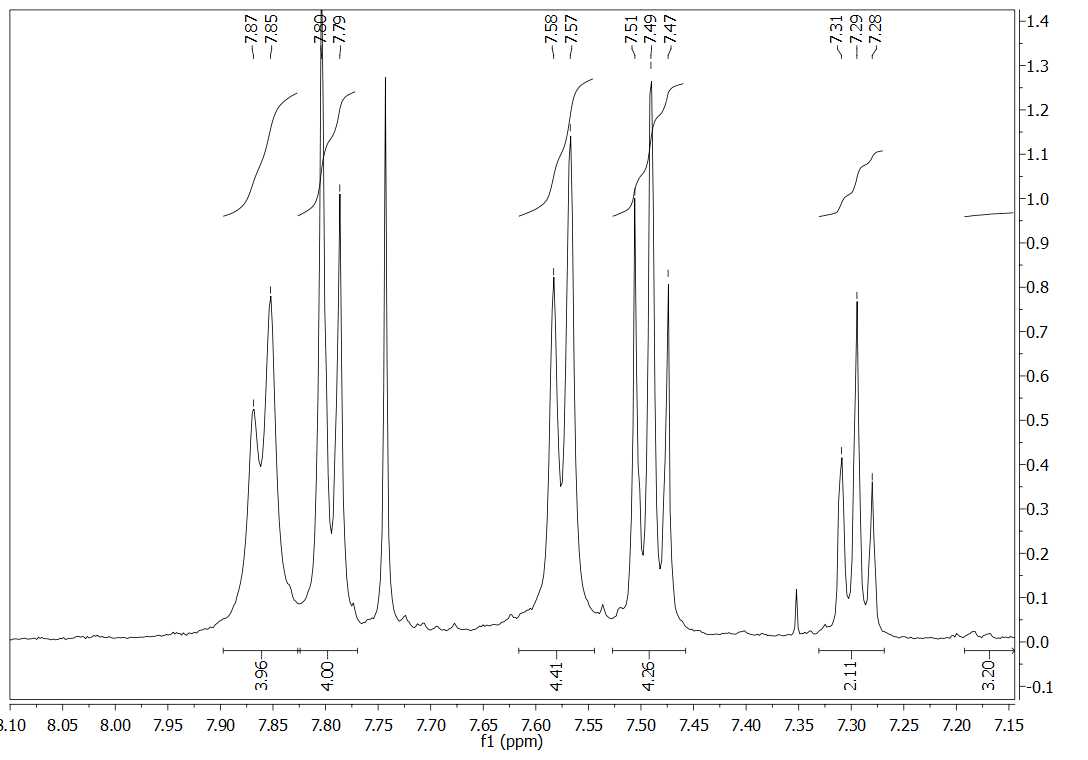


Figure S19.^1^H NMR (DMSO-*d6*, 500 MHz) spectrum of 4,4'-([1,1'-biphenyl]-4,4'-diylbis(diazene-2,1-diyl))bis(1-phenyl-1*H*-pyrazole-3,5-diamine) **7**


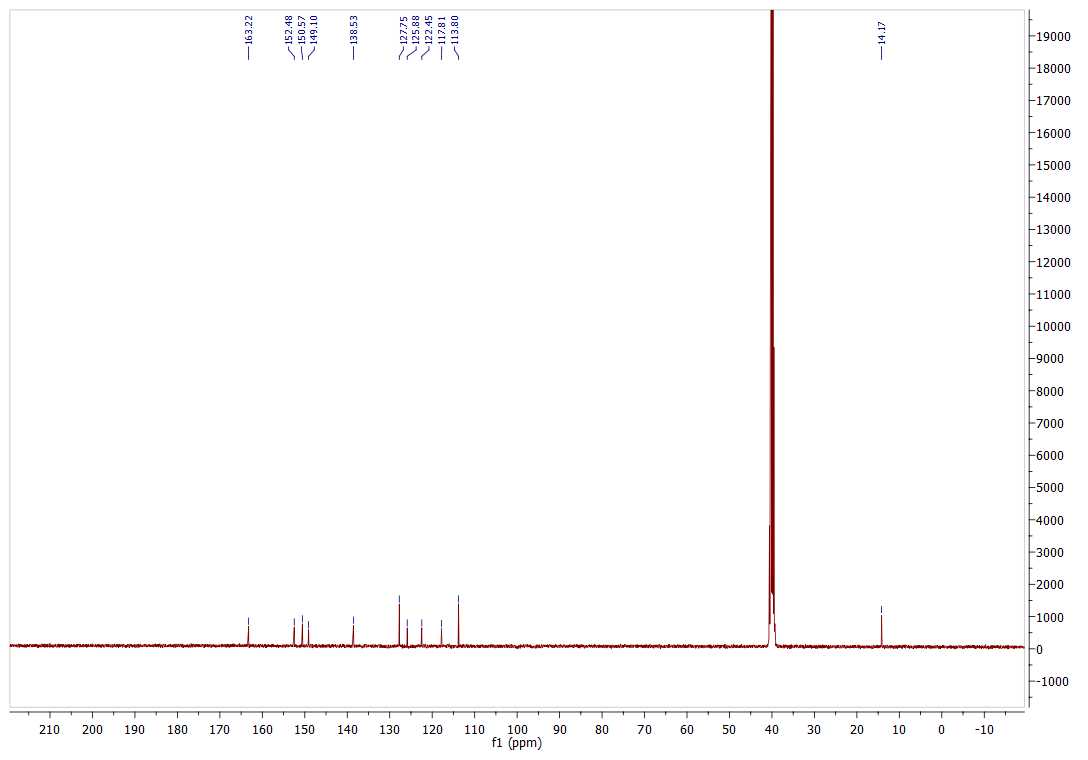


Figure S20. ^13^C NMR (DMSO-*d6*, 500 MHz) spectrum of 4,4'-([1,1'-biphenyl]-4,4'-diylbis(diazene-2,1-diyl))bis(1-phenyl-1*H*-pyrazole-3,5-diamine) **7**

Figure S21. Optimized geometry of dyes under investigation

| 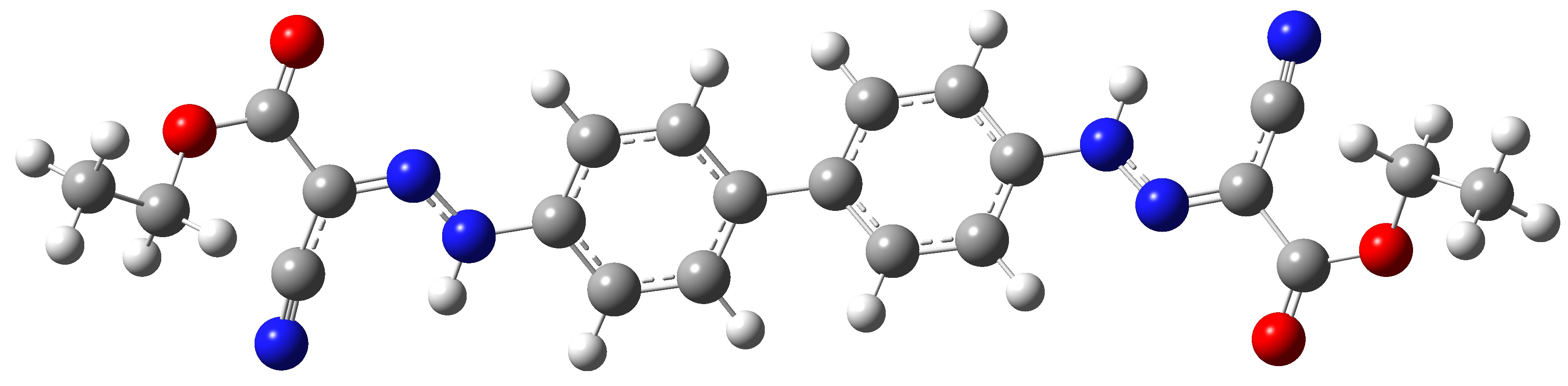 | 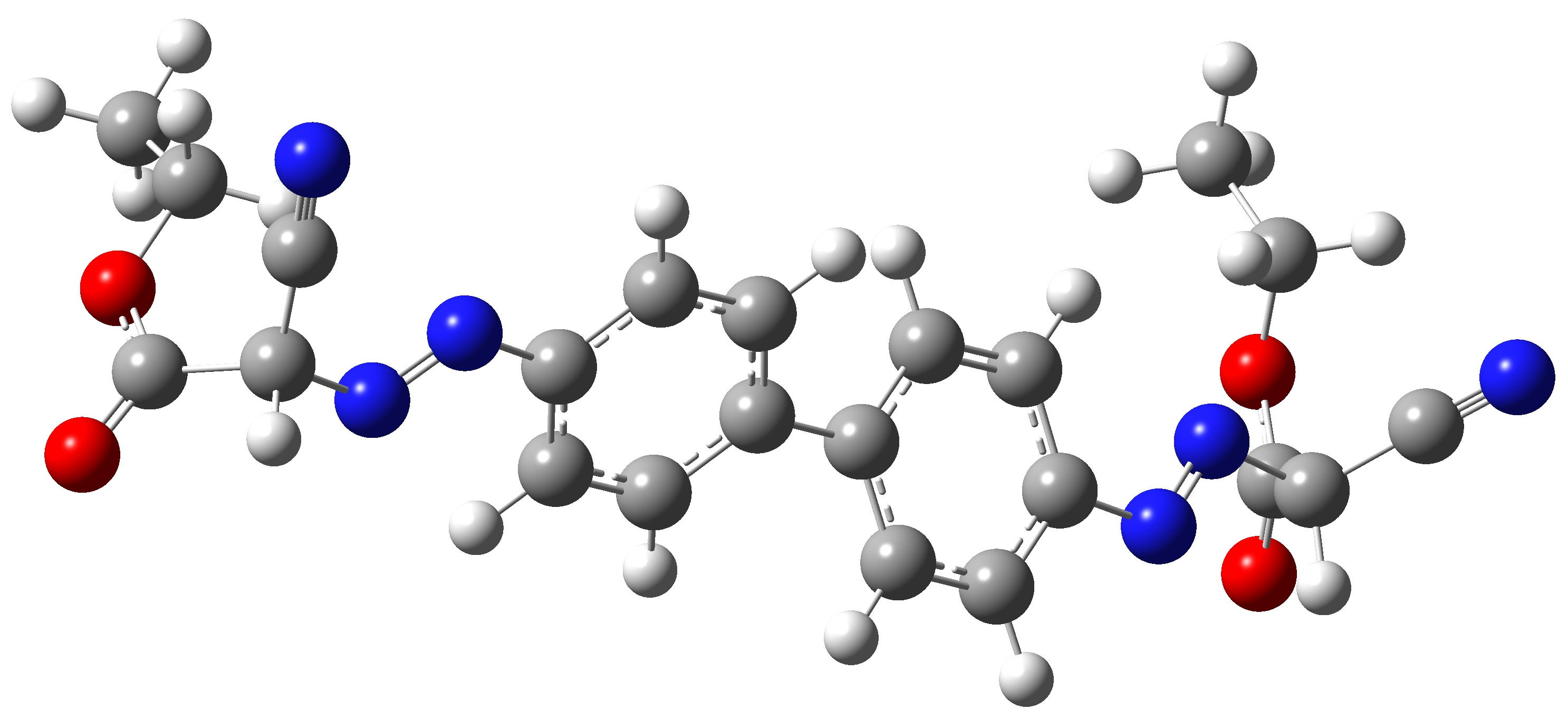 |
| --- | --- |
| 3 Hydrazone tautomer | 3 Azo tautomer |
| 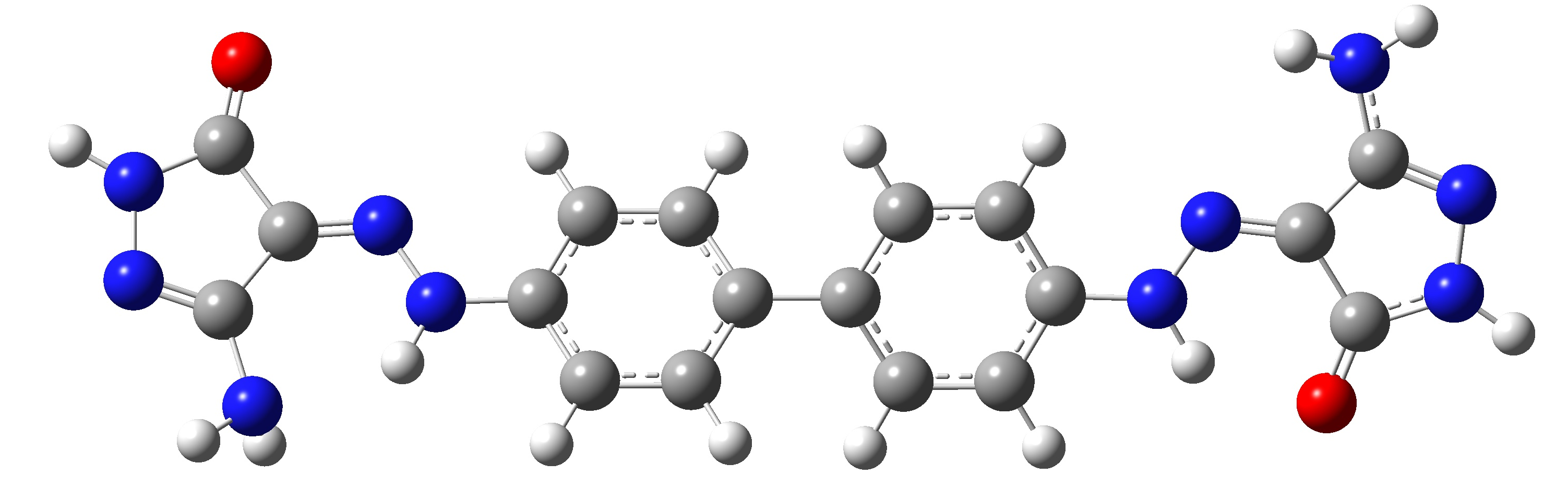 | 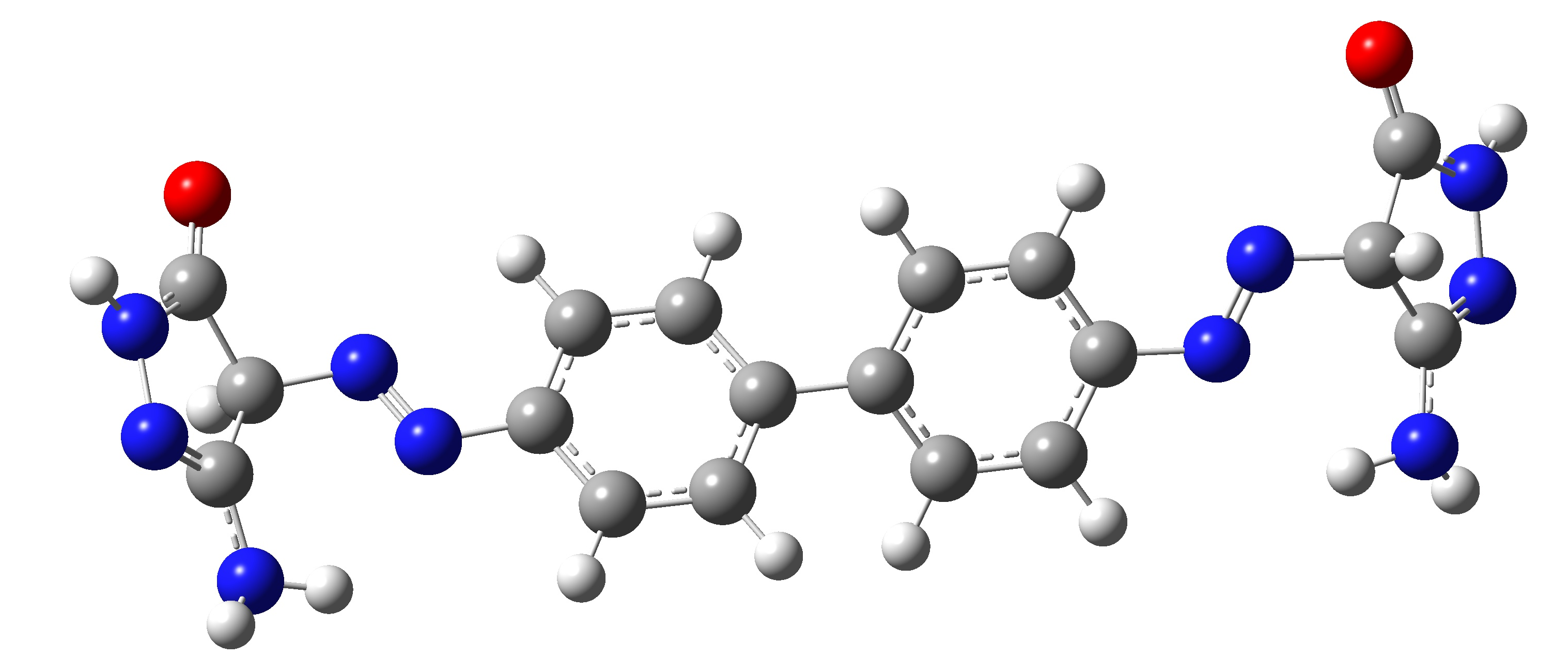 |
| 5a Hydrazone tautomer | 5a Azo tautomer |
| 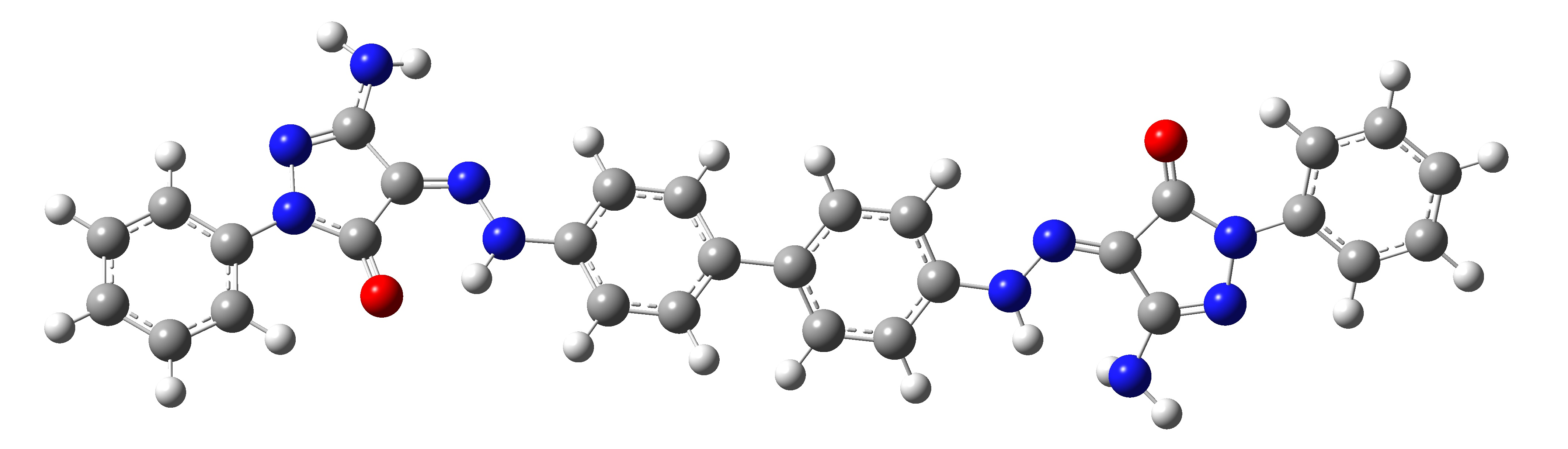 | 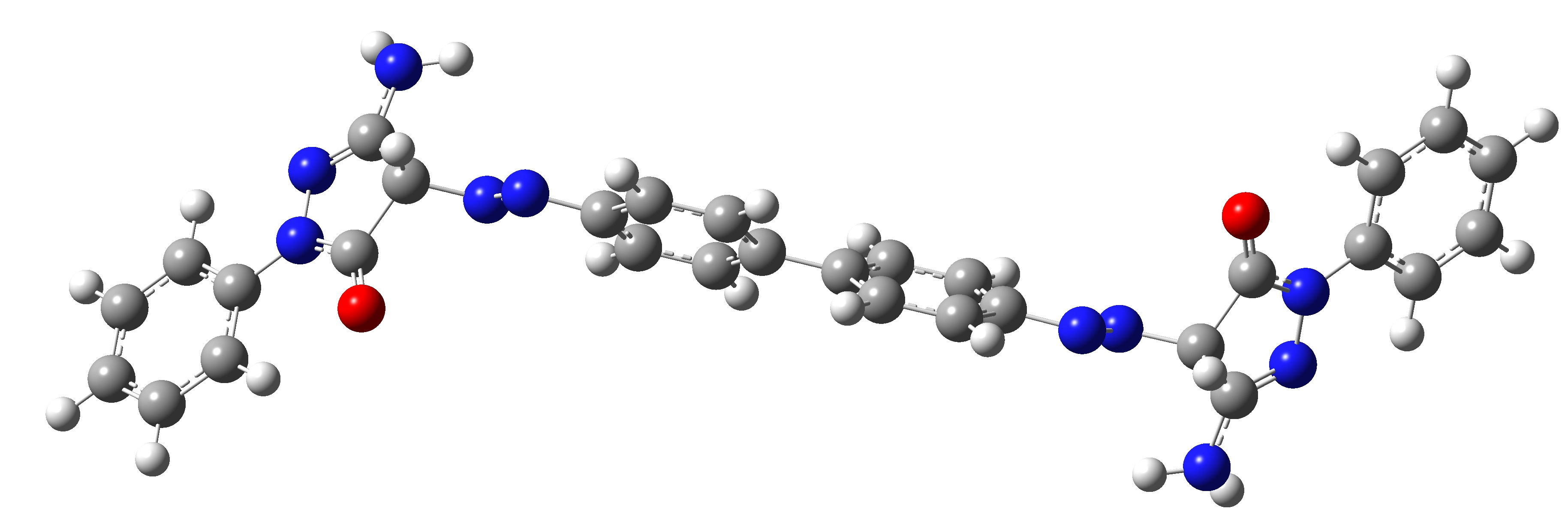 |
| 5b Hydrazone tautomer | 5b Azo tautomer |
| 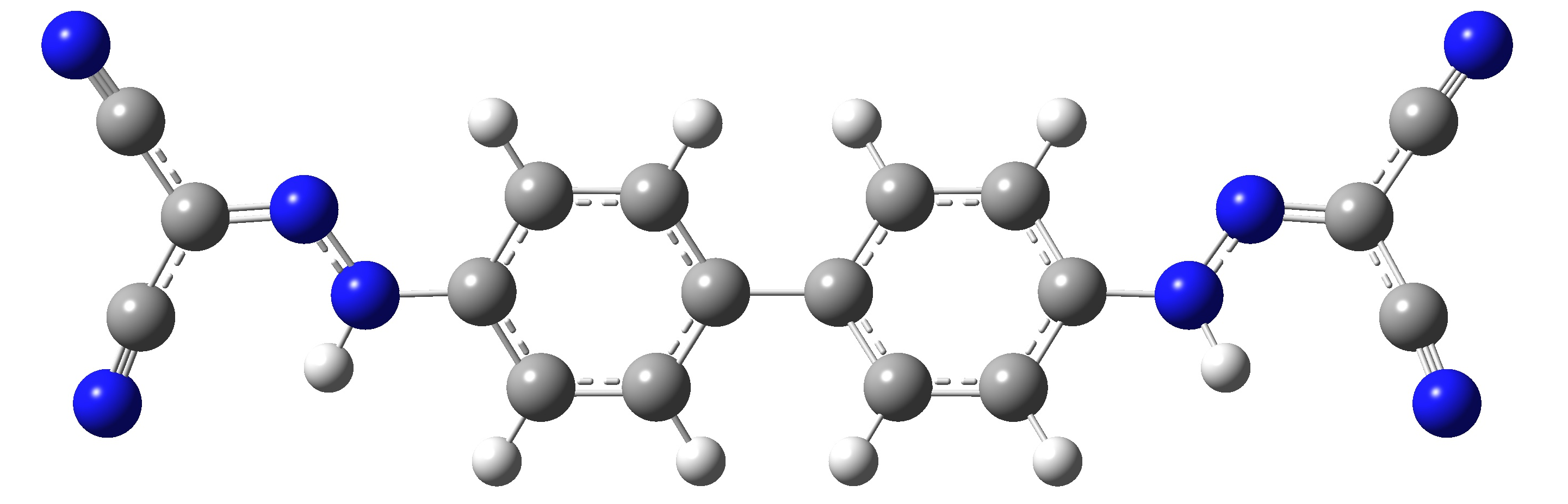 | 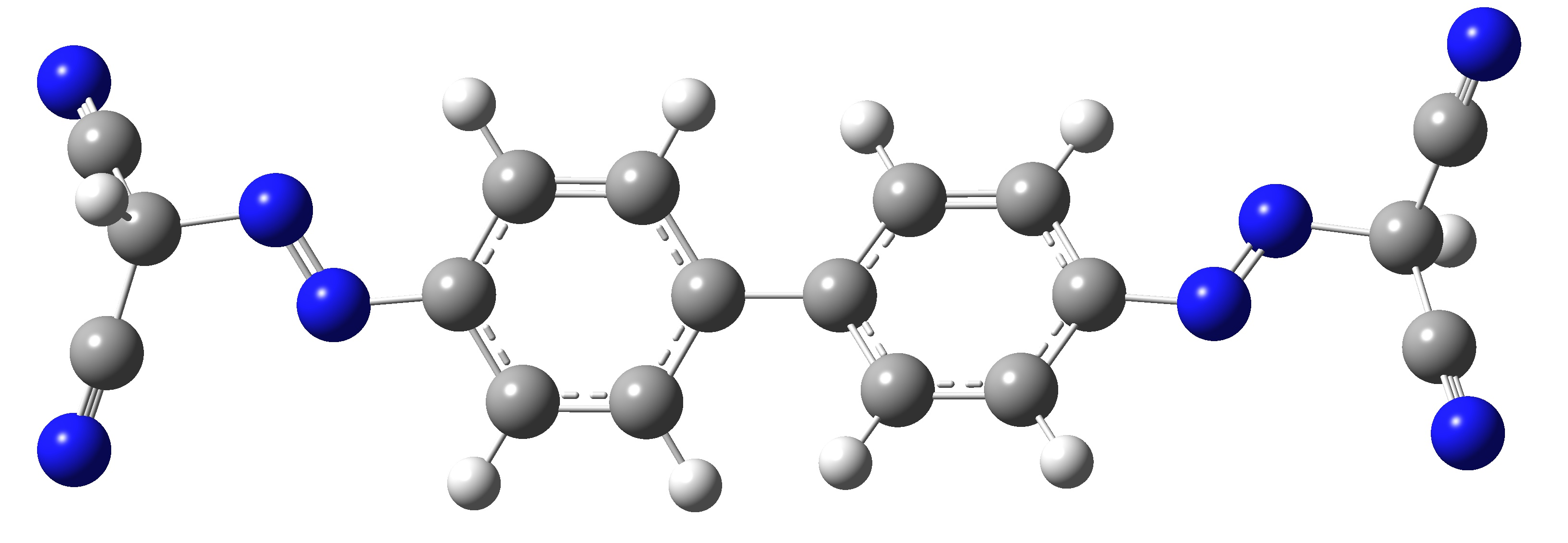 |
| 4 Hydrazone tautomer | 4 Azo tautomer |
| 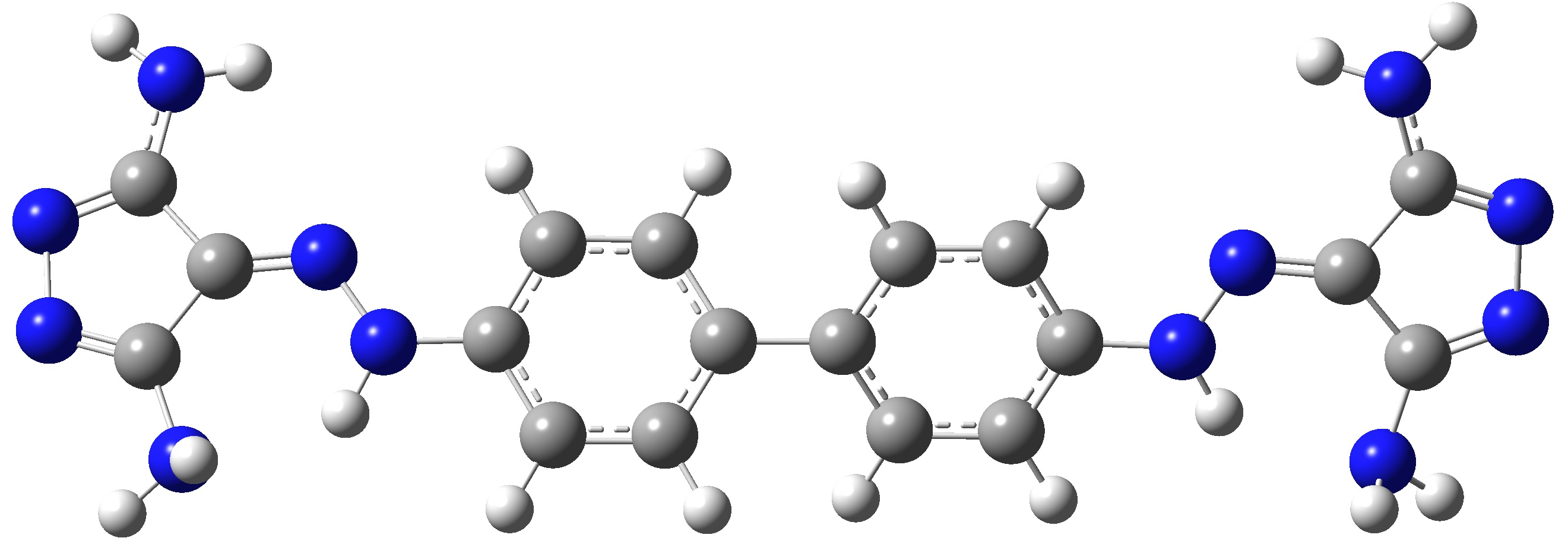 | 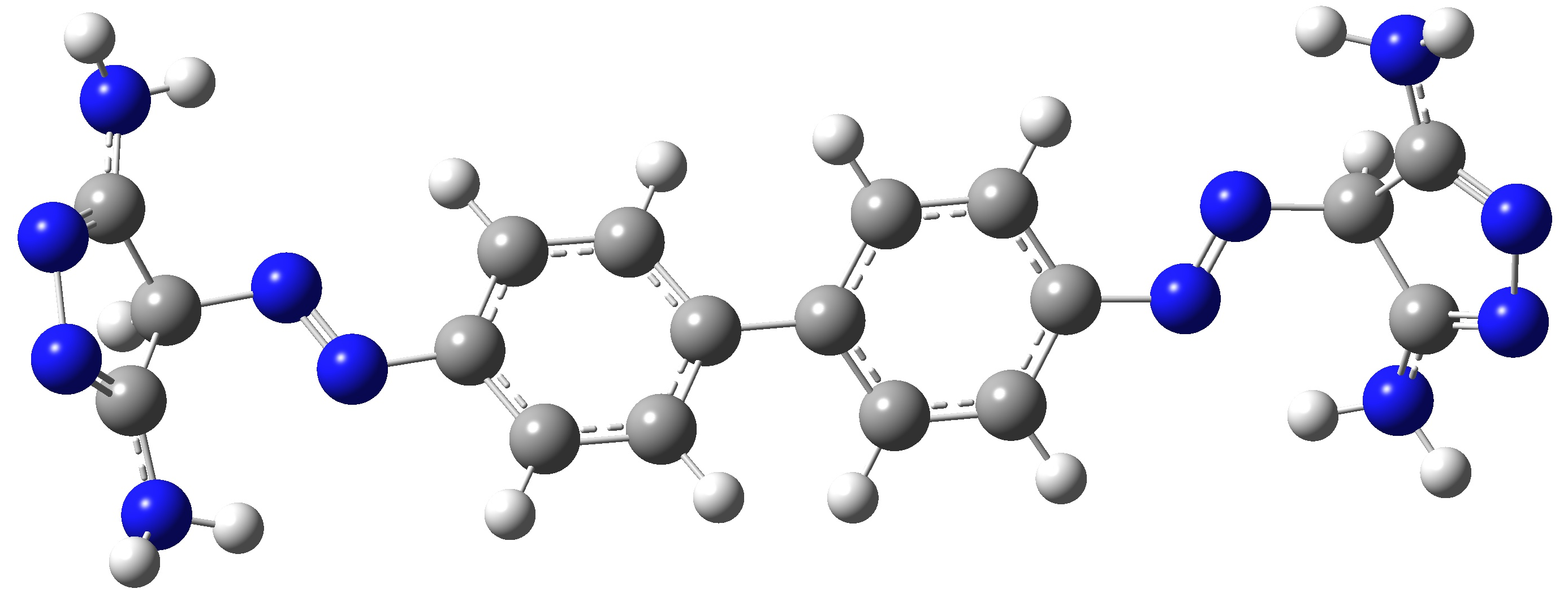 |
| 6 Hydrazone tautomer | 6 Azo tautomer |
| 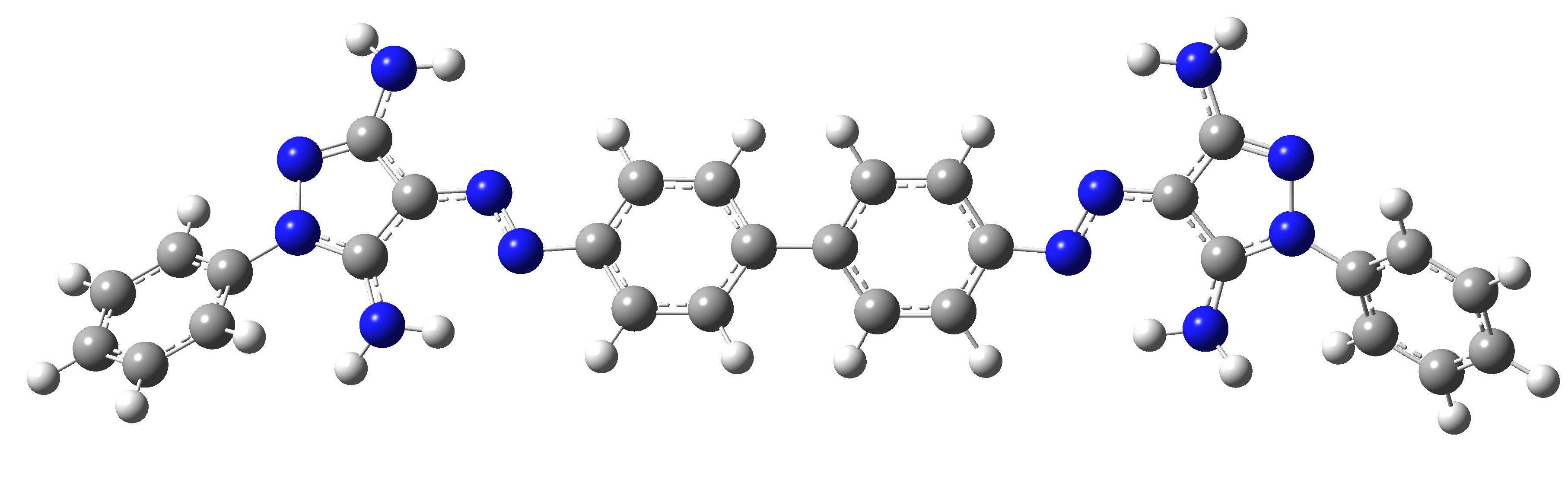 | |
| 7 Azo tautomer | |
